# Endolysosomal inhibition uncouples tau uptake from intracellular seeding

**DOI:** 10.1016/j.jbc.2026.113498

**Published:** 2026-08-27

**Authors:** Dana A. Dodd, Michael S. LaCroix, Clarissa Valdez, Ayde Mendoza-Oliva, Joshua D. Beaver, Anthony R. Vega, Ashwani Kumar, Chao Xing, Charles L. White, Marc I. Diamond

**Affiliations:** 1 Center for Alzheimer’s and Neurodegenerative Diseases, Peter O’Donnell Jr. Brain Institute, University of Texas Southwestern Medical Center, Dallas, Texas, USA; 2 Eugene McDermott Center for Human Growth and Development, University of Texas Southwestern Medical Center, Dallas, Texas, USA; 3 Department of Bioinformatics, University of Texas Southwestern Medical Center, Dallas, Texas, USA; 4 Department of Population and Data Science, University of Texas Southwestern Medical Center, Dallas, Texas, USA; 5 O’Donnell School of Public Health, University of Texas Southwestern Medical Center, Dallas, Texas, USA; 6 Department of Pathology, University of Texas Southwestern Medical Center, Dallas, Texas, USA

**Keywords:** tauopathy, Alzheimer’s disease, endocytosis, vacuolar ATPase, Rab5a, tau seeding

## Abstract

Neurodegenerative tauopathies, including Alzheimer’s disease, appear to be driven by propagation of tau assemblies, which must access the cytoplasm to recruit monomer and self-replicate, a process termed “seeding.” The prevailing model holds that tau seeds enter cells *via* macropinocytosis and reach the cytosol through lysosomal rupture or microperforation. Our findings revise this model by revealing that endocytosis is not required for seeding. Using genome-scale clustered regularly interspaced short palindromic repeats screening, we identified multiple vacuolar H^+^-ATPase components whose loss reduced tau uptake (measured by flow cytometry) yet paradoxically increased seeding (measured by FRET biosensors). Acute vacuolar H^+^-ATPase inhibition with bafilomycin A1 produced the same effect in v2L tau biosensors and induced pluripotent stem cell-derived neurons. Among regulators of endosome maturation, dominant-negative Rab5a decreased internalization while enhancing cytoplasmic seeding. Cholesterol depletion produced identical results. Strikingly, transient hypothermia eliminated virtually all detectable tau uptake and dramatically increased seeding, without affecting subsequent tau monomer or aggregate degradation. We conclude that efficient endolysosomal trafficking does not appear to be required for cytoplasmic seeding under the conditions studied here. Across diverse perturbations, reduced endolysosomal flux consistently enhanced tau seeding, consistent with prior work indicating that most internalized aggregates are routed toward degradation rather than amplification. To seed effectively, tau must cross the plasma or vesicular membranes into the cytoplasm. We have found that proper endolysosomal trafficking suppresses cytoplasmic tau seeding, as all perturbations augmented this process. These findings reframe the role of the endolysosomal system in tau seeding and identify membrane transit into the cytoplasm, rather than macropinocytosis itself, as a critical gateway to intracellular replication.

Tau is a thermostable, intrinsically disordered microtubule binding protein (1). Neurodegenerative tauopathies, including Alzheimer’s disease (AD), feature progressive accumulation of tau aggregates within neurons and glia (2). In these disorders, tau monomer forms pathological assemblies that propagate in stereotyped patterns along large-scale functional neuronal networks (3, 4, 5). A substantial body of experimental and observational data support a prion model in which distinct tau strains, or self-propagating fibrillar assemblies of defined conformation, move between adjacent cells to serve as templates that recruit tau monomer, a process termed “seeding” (6). Different strains cause distinct neuropathological phenotypes in experimental mice (7), which could explain the diversity of clinical phenotypes in patients.

Our lab originally observed that exogenous tau assemblies efficiently enter cells and trigger intracellular aggregation of endogenous tau monomer (8). Tau uptake requires binding to specific heparan sulfate proteoglycans (HSPGs) motifs, which initiate macropinocytosis and internalization of tau assemblies (9, 10). Soluble HSPG mimetics such as heparin inhibit tau binding, and genetic disruptions of HSPG synthesis or modification block both uptake and seeding (9, 10). Despite nearly universal cellular uptake of recombinant fibrils, intracellular seeding is typically inefficient, affecting less than 10% of cells after exposure (11). Our prior work indicates that most internalized seeds are rapidly degraded in the lysosome, while only a small fraction gain access to the cytoplasm where clearance occurs *via* the proteasome (11). Importantly, uptake and seeding are experimentally separable processes.

It is unclear how pathogenic tau accesses the cytoplasm after endocytosis. Vesicle rupture has been proposed as a principal mechanism (12), but recent reports suggest that overt membrane damage induced by fibrils is rare and seeds instead cross membranes through transient nanoscale disruptions (11, 13, 14). Thus, the relative contributions to tau cytoplasmic seeding of macropinocytosis, endolysosomal maturation, vesicle damage, and membrane permeabilization remain unresolved.

We have developed methods to quantify tau uptake using flow cytometry (9), and to measure induction of intracellular aggregation based on FRET in engineered “biosensor” cell lines (15). The most recent tau biosensor, termed v2L, features stable expression of the tau repeat domain (RD) with the disease-associated P301S mutation fused to complementary fluorescent proteins, cerulean and clover (16). FRET flow cytometry measures induction of aggregation in thousands of cells per condition, rendering it very accurate at measuring seeding activity.

To define the cellular pathways that govern tau uptake, we began by performing a genome-wide clustered regularly interspaced short palindromic repeats (CRISPR)-Cas9 screen in human embryonic kidney 293 T (HEK293T) cells. This analysis revealed multiple components of the vacuolar H + -ATPase (v-ATPase) complex that are essential for endosomal acidification and maturation as regulators of tau uptake. This was unexpected given the canonical role of the v-ATPase in vesicle acidification rather than cargo trafficking. Follow-up studies in tau biosensor cells and human induced pluripotent stem cell (iPSC)-derived neurons uncovered a consistent and surprising inverse correlation: perturbations that reduced or blocked endocytosis, including v-ATPase inhibition, dominant-negative Rab5a expression, cholesterol depletion, or transient hypothermia, each markedly enhanced intracellular tau seeding.

These findings challenge the prevailing model that a functional endocytic pathway is required for tau seeds to reach the cytoplasm. Instead, they support a revised framework in which efficient endolysosomal trafficking protects cells by routing seeds for degradation, whereas slowing or even completely blocking trafficking allows tau assemblies to traverse cellular membranes and initiate cytoplasmic seeding.

## Results

### v-ATPase function is required for tau uptake

To identify cellular proteins involved in tau internalization, we performed a genome-wide CRISPR-Cas9 loss-of-function screen in HEK293T cells (17). Uptake of tau fibrils is mediated by macropinocytosis (9) and is very slow in comparison to classical receptor-mediated uptake of molecules such as transferrin (Fig. 1*A*). We therefore performed the screen at a 4 h timepoint. We confirmed recombinant tau fibril formation by transmission electron microscopy (Fig. S1*A*), and empirically determined an optimal concentration for uptake of 25 nM (monomer equivalent) (Fig. 1*B*). After tau fibril-647 exposure we sorted Alexa647 negative cells in two phases, gating according to signal from control cells (Figs. 1*C* and S2, *A*–*C*).

**Figure 1:**
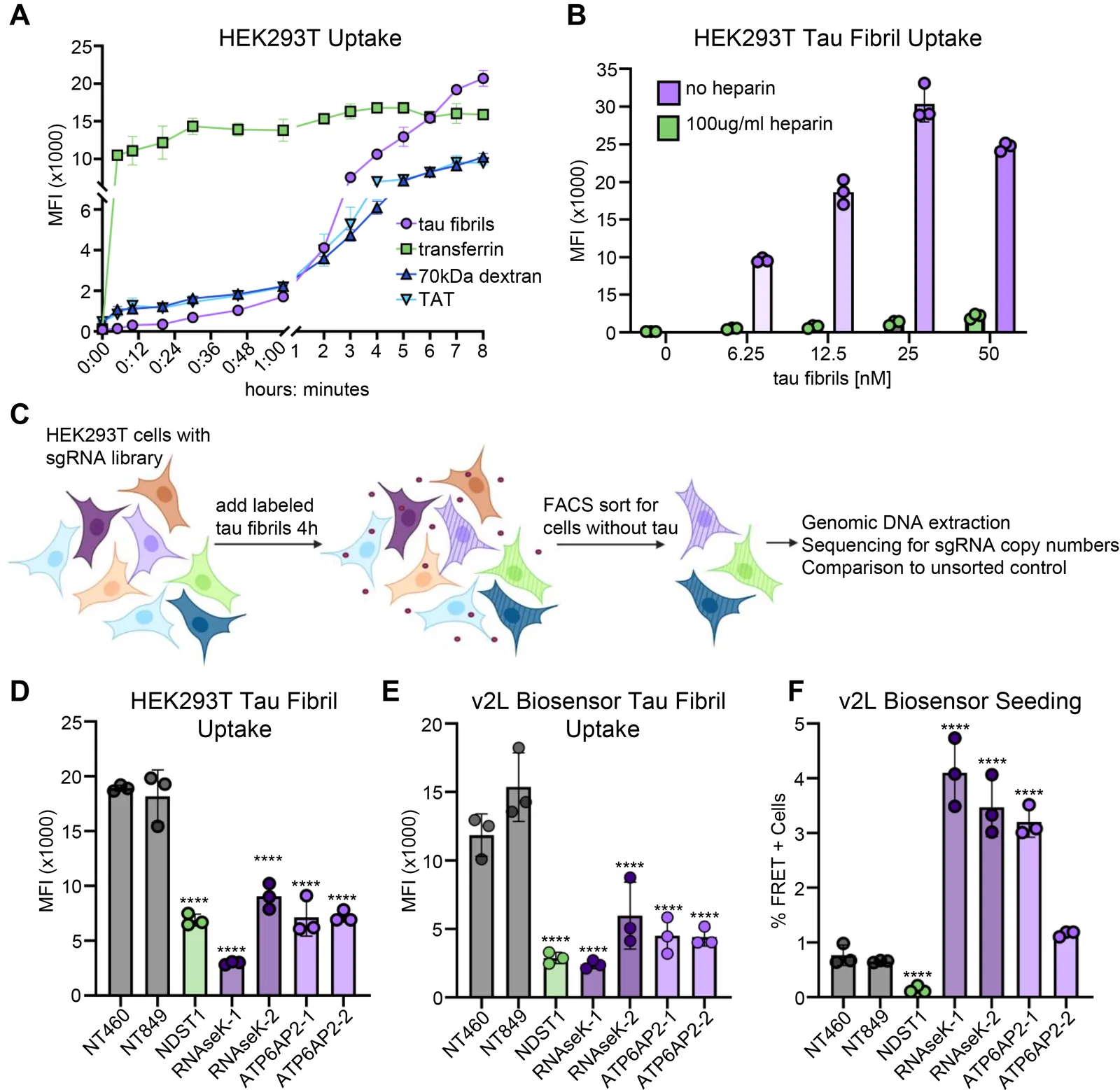
**CRISPR screen identifies regulators of tau fibril uptake and seeding.***A*, HEK293T cells were incubated for the indicated times with Alexa647-labeled tau fibrils (25 nM), transferrin (250 nM), TAT peptide (5 μM), or dextran–Texas Red (1.4 μM). Cells were treated with 0.25% trypsin to remove surface-bound material and analyzed by flow cytometry. Uptake is reported as median fluorescence intensity (MFI). *B*, HEK293T cells were incubated for 4 h with increasing concentrations of Alexa647-labeled tau fibrils in the presence or absence of heparin (100 μg/ml). Following trypsin treatment, intracellular tau was quantified by flow cytometry. *C*, v2L tau biosensor cells were transduced with the GeCKO v2 sgRNA lentiviral library at a multiplicity of infection (MOI) of 0.3 and exposed to Alexa647-labeled tau fibrils for 4 h. Alexa647-negative cells were isolated by fluorescence-activated cell sorting, sgRNAs were sequenced, and enrichment was determined relative to unsorted cells. *D* and *E*, HEK293T cells (*D*) or v2L biosensor cells (*E*) were transduced with lentivirus encoding nontargeting sgRNAs (NT460 and NT849) or individual sgRNAs targeting genes identified in the screen. Cells were incubated with Alexa647-labeled tau fibrils (25 nM) for 4 h, treated with trypsin, and tau uptake was quantified by flow cytometry. *F*, v2L biosensor cells expressing nontargeting or gene-specific CRISPR knockout sgRNAs were incubated with tau fibrils (50 nM) for 48 h. Tau aggregation was quantified as the percentage of FRET-positive cells by flow cytometry. All experiments were performed at least twice with similar results. Data represent mean ± SD of technical replicates. Statistical analysis was performed using one-way ANOVA with Dunnett’s multiple-comparisons test compared to NT849; ∗∗∗∗*p* < 0.0001. CRISPR, clustered regularly interspaced short palindromic repeats; HEK293T, human embryonic kidney 293 T; sgRNA, single guide RNA; TAT, trans-activator of transcription.

Deep sequencing of sgRNAs from tau uptake-negative cells revealed 963 significant hits (*p* < 0.05) (Table S1). In the GeCKO library, each gene is targeted by six gRNAs, and 74% of the top 963 included all six independent sgRNAs. Within the hits were multiple expected regulators of HSPG synthesis such as NDST1, which we previously determined to be critical for tau aggregate uptake and seeding (9, 10), and 6 other genes involved in HSPG synthesis. Unexpectedly, several of the strongest hits were components of the v-ATPase, including RNAseK- the top hit, ATP6AP2, ATP6V0A2, ATP6V2G1, ATP6V1A, and ATP6V0D2. The v-ATPase is essential for acidification and maturation of endosomal and lysosomal compartments (18). Additional hits in RAB GTPases, SNARE proteins, ESCRT machinery, and membrane lipid metabolism genes were also consistent with endolysosomal-dependent membrane transit. A comprehensive pathway-level annotation of screen hits is provided in Table S2.

### Genetic inhibition of v-ATPase decreases tau uptake and increases seeding

To test the screening results, we generated CRISPR KO in HEK293T and tau biosensor cells targeting RNAseK and ATP6AP2 (9, 10). We confirmed RNAseK and ATP6AP2 KO by inference of CRISPR edits (Fig. S3) (19). As predicted, disruption of either gene significantly reduced tau uptake relative to nontargeting controls, comparable to the loss of NDST1 (Fig. 1, *D* and *E*). We determined an optimal concentration of tau fibrils for seeding to be 50 nM (monomer equivalent) (Fig. S1*B*). Seeding was measured by FRET flow cytometry with v2L tau biosensors (16). Unexpectedly, KO of RNAseK or ATP6AP2 markedly increased tau seeding (Fig. 1*F*). NDST1 KO decreased both uptake and seeding, which was expected because, unlike vesicle transport regulators, NDST1 is required for tau binding to HSPGs on the plasma membrane (16). These data suggested that endolysosomal trafficking might restrict, rather than promote, access of tau seeds to the cytosol.

### Bafilomycin decreases tau uptake and increases seeding

To further test the role of the v-ATPase, we used bafilomycin A1, a potent and reversible inhibitor (20). Pretreatment of HEK293T cells or human iPSC-derived neurons (Fig. S4, *A* and *B*) with varying concentrations of bafilomycin for 0.5 h prior to tau exposure reduced tau uptake (Fig. 2, *A* and *C*). Pretreatment of cells with bafilomycin for 1 h without tau fibrils, followed by washing and tau fibril addition without any bafilomycin also reduced tau uptake and increased tau seeding (Fig. S5, *A* and *B*). Bafilomycin increased seeding robustly and dose-dependently in both HEK293T and iPSC-derived neurons (Figs. 2, *B*, *D* and S6). Remarkably, even a 30 min pretreatment with bafilomycin followed by washout was sufficient to reduce uptake and increase seeding (Fig. 2*B*). To exclude the possibility that tau was trapped at the cell surface and subsequently trafficked into the cell after bafilomycin was removed, we treated cells with tau and bafilomycin followed by trypsin exposure sufficient to digest extracellular tau (9), and still observed increased seeding (Fig. 2, *E* and *F*), consistent with facilitation of tau seed entry directly into the cytoplasm by inhibition of internalization. Thus, interference of endolysosomal uptake of tau fibrils markedly enhanced tau seeding.

**Figure 2:**
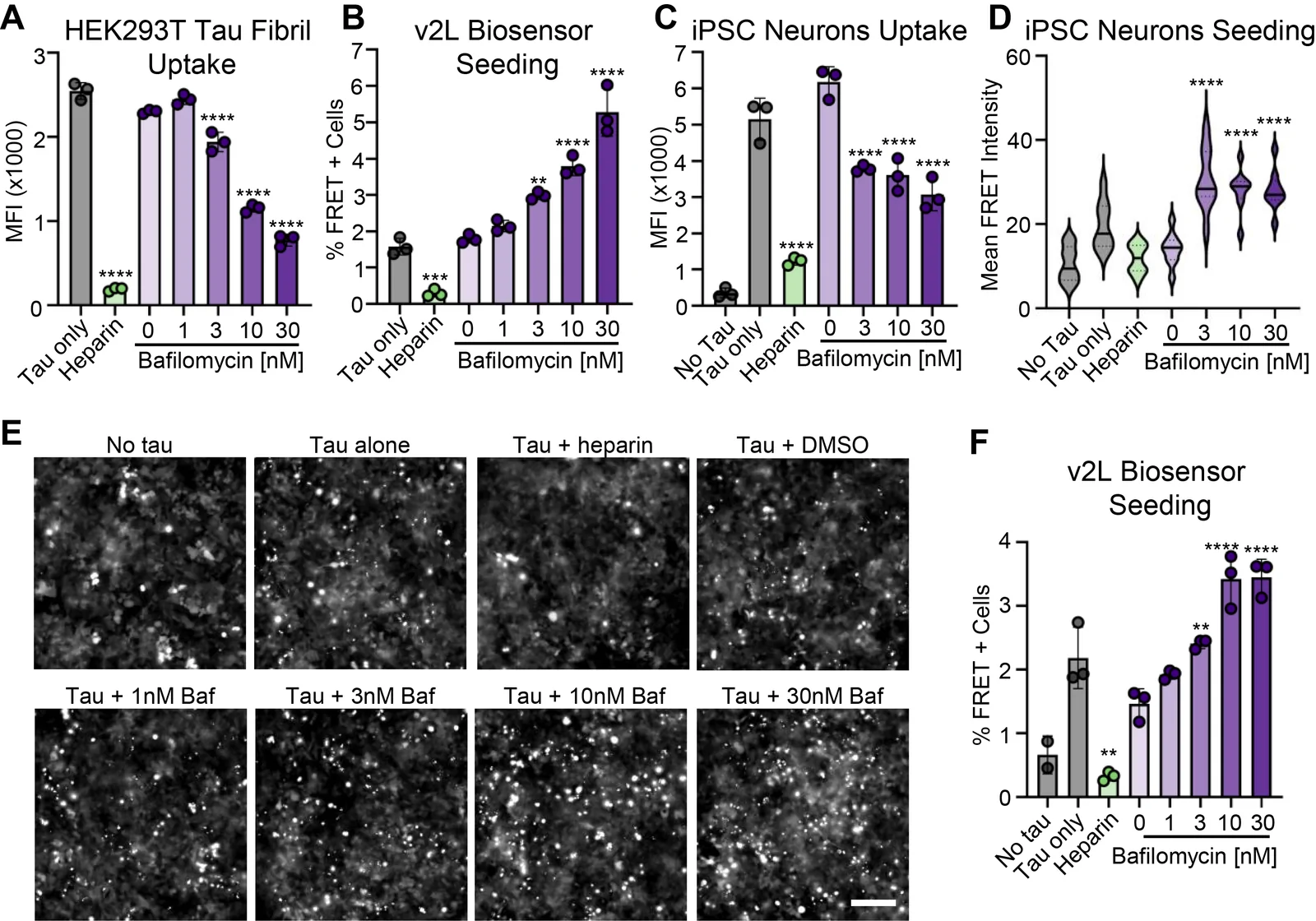
**Inhibition of endolysosomal acidification reduces tau uptake but enhances intracellular seeding.***A*, HEK293T cells were pretreated with the indicated concentrations of bafilomycin A1 for 30 min, followed by incubation with sonicated Alexa647-labeled tau fibrils (25 nM) in the presence of bafilomycin for 4 h. Cells were treated with trypsin to remove surface-bound tau, and intracellular uptake was quantified by flow cytometry as median fluorescence intensity (MFI). *B*, v2L tau biosensor cells were pretreated with bafilomycin for 30 min followed by washout, and incubated with tau fibrils for 4 h in the continued presence of compound. Heparin (200 μg/ml) was included as a control to block tau binding and uptake. Cells were washed, incubated for 48 h, and intracellular tau aggregation was quantified by FRET flow cytometry. *C*, iPSC-derived neurons were incubated with Alexa647-labeled tau fibrils (25 nM) and bafilomycin A1 for 4 h, washed, and incubated for an additional 48 h prior to quantification of tau aggregation by FRET flow cytometry. *D*, iPSC-derived neurons expressing tau-RD-P301S-Clover and tau-RD-P301S-Ruby were generated by lentiviral transduction. Four days posttransduction, neurons were incubated with sonicated tau fibrils (20 nM) and bafilomycin for 4 h. Heparin (200 μg/ml) served as a control to block tau binding and uptake. After washing to remove bafilomycin, neurons were incubated for 48 h, and FRET intensity was quantified by high-content imaging. *E*, v2L biosensor cells were incubated with tau fibrils and bafilomycin for 4 h, with heparin as a positive control, treated with trypsin (0.25%, 5 min, 37 °C), replated without bafilomycin, and imaged 48 h later using an ImageXpress system (Molecular Devices). The scale bar represents 150 μm. *F*, cells from *E* were harvested and analyzed by flow cytometry to quantify FRET. All experiments were performed at least twice with similar results. Data represent mean ± SD of technical replicates. Statistical significance was assessed by one-way ANOVA with Dunnett’s multiple-comparisons test compared to 0 nM bafilomycin for *A*, *B*, *C*, and *F*, and by two-way ANOVA with Dunnett’s multiple-comparisons test compared to 0 nM bafilomycin for *D*. ∗∗*p* < 0.01, ∗∗∗*p* < 0.001, ∗∗∗∗*p* < 0.0001. HEK293T, human embryonic kidney 293 T; iPSC, induced pluripotent stem cell; RD, repeat domain.

### Disruption of endosomal maturation decreases tau uptake and increases seeding

We next tested whether other perturbations of endosomal maturation produced similar effects on tau internalization and seeding. To directly interfere with endocytic trafficking, we overexpressed dominant-negative regulators of distinct endocytic pathways using lentiviral vectors that coexpress TagRFP. Rab5 and Rab7 both control macropinocytosis and clathrin-mediated endocytosis, with Rab5 acting at early endosomes and Rab7 at late endosomes (21, 22). Caveolin and dynamin control caveolae-mediated endocytosis, whereas Arf6 and Rac1 mediate Arf6-mediated endocytosis (23, 24). Cdc42 controls GRAF1-dependent endocytosis (25). Eps15 is a protein essential for clathrin-mediated endocytosis (26). All constructs expressed TagRFP and many changed cellular morphology. However, only dominant negative Rab5a(S34N) significantly reduced tau uptake (Fig. 3*A*) and increased seeding by ∼40-fold (Fig. 3*B*). These findings indicated that impaired early endosomal formation or trafficking enhanced tau seeding despite reduced internalization of tau assemblies.

**Figure 3:**
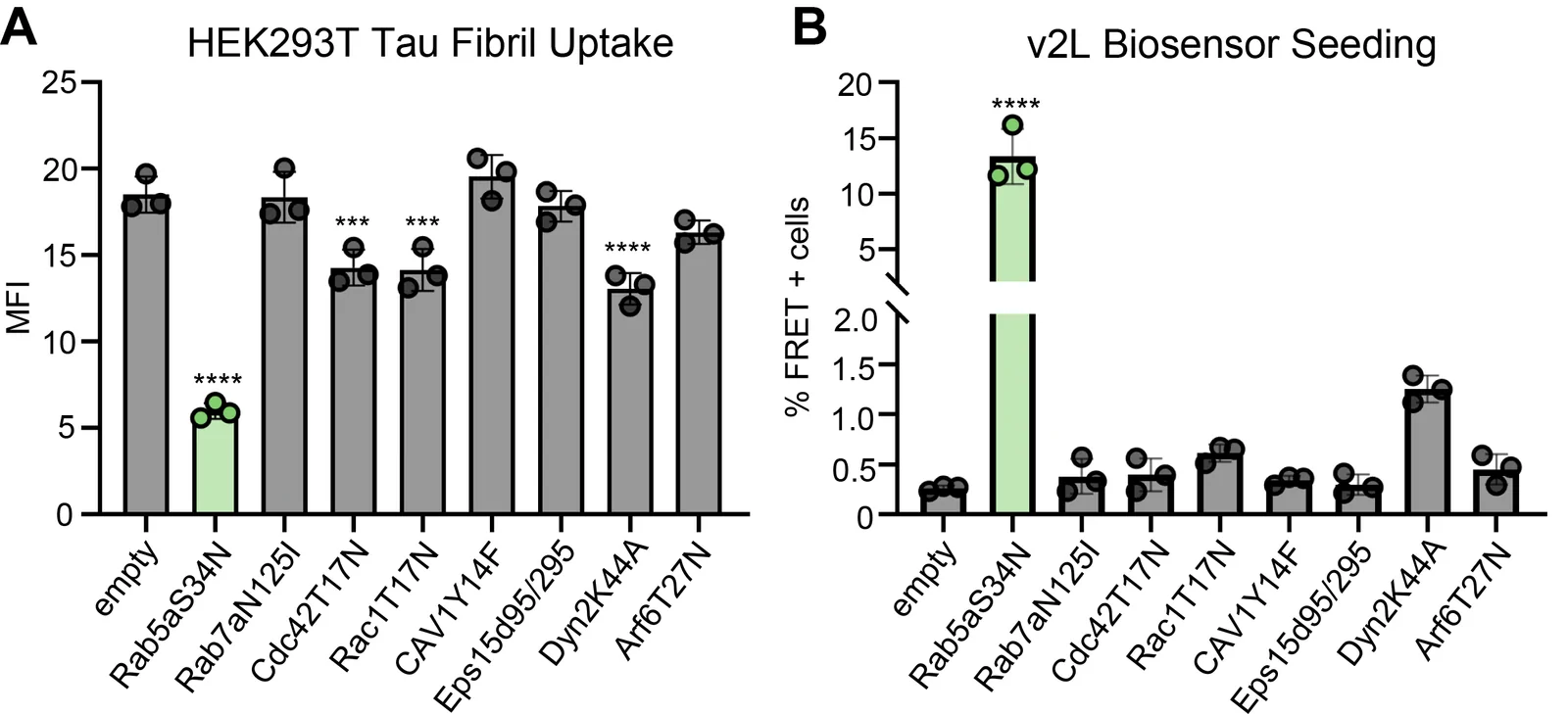
**Dominant-negative Rab5a expression reduces tau uptake and enhances seeding.***A*, HEK293T cells were transduced with lentivirus expressing TagRFP alone (empty vector) or TagRFP coexpressing dominant-negative Rab5a. Four days posttransduction, cells were incubated with sonicated Alexa647-labeled tau fibrils (25 nM) for 4 h. Following trypsin treatment to remove surface-bound tau, intracellular tau uptake was quantified by flow cytometry. *B*, v2L tau biosensor cells were transduced with the same lentiviral constructs described in *A*. Four days posttransduction, cells were incubated with tau fibrils (50 nM) for 48 h, after which intracellular tau aggregation was quantified by FRET flow cytometry. All experiments were performed at least twice with similar results. Data represent mean ± SD of technical replicates. Statistical analysis was performed using one-way ANOVA with Dunnett’s multiple-comparisons test compared to empty. ∗∗∗*p* < 0.001, ∗∗∗∗*p* < 0.0001. HEK293T, human embryonic kidney 293 T.

### Cholesterol depletion increases seeding

Cholesterol depletion by methyl-beta cyclodextrin (MBCD) has been reported to reduce (27) and increase (28) macropinocytosis, and has previously been reported to increase tau entry into the cytoplasm (29). We therefore used MBCD at confirmed nontoxic doses to deplete cellular cholesterol (Fig. S7) to test effects on tau uptake and seeding. We incubated cells for 0.5 h with increasing amounts of MBCD prior to 4 h of fibril treatment along with MBCD. MBCD dose-dependently reduced tau uptake in HEK293T cells (Fig. 4*A*) and increased seeding in v2L biosensor cells (Fig. 4*B*). These results paralleled the effects of v-ATPase inhibition and dominant-negative Rab5a, consistent with slowed or impaired vesicle trafficking as an enhancer of cytosolic tau seeding.

**Figure 4:**
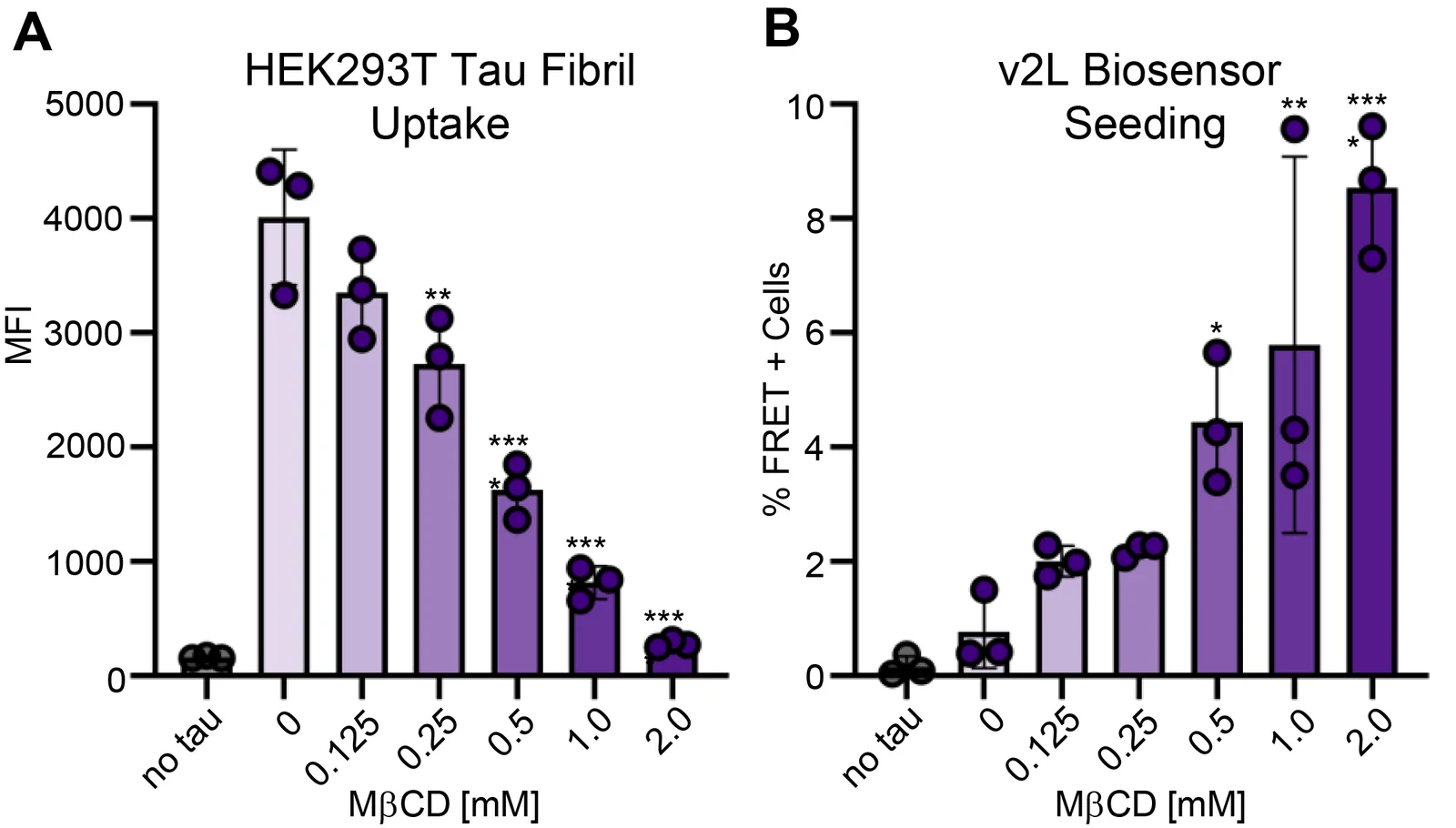
**Cholesterol depletion reduces tau uptake and enhances intracellular seeding.** Cells were plated 1 day prior to experimentation. Methyl-β-cyclodextrin (MβCD) was prepared fresh on the day of the experiment. Culture medium was completely removed and replaced with serum-free medium containing the indicated concentrations of MβCD for 30 min. *A*, HEK293T cells were incubated with Alexa647-labeled tau fibrils (25 nM) for 4 h following MβCD treatment. Cells were harvested, treated with trypsin to remove surface-bound tau, and tau uptake was quantified by flow cytometry. *B*, v2L tau biosensor cells were incubated with tau fibrils (50 nM) for 4 h following MβCD treatment, then trypsinized, replated, and incubated at 37 °C for 48 h prior to quantification of intracellular tau aggregation by FRET flow cytometry. All experiments were performed at least twice with similar results. Data represent mean ± SD of technical replicates. Statistical analysis was performed using one-way ANOVA with Dunnett’s multiple-comparisons test compared to 0 nM MBCD; ∗*p* < 0.05, ∗∗*p* < 0.005, ∗∗∗∗*p* < 0.0001.

### Transient hypothermia blocks endocytosis and increases tau seeding

The preceding experiments indicated that inhibiting endolysosomal function and maturation, while blocking tau uptake, paradoxically increased seeding. Transient hypothermia, at temperatures at or below 10 °C, globally and reversibly blocks all endocytosis (30, 31). To test whether tau seeding could occur when endocytosis was halted, we exposed cells to 4 °C or 10 °C during tau fibril treatment. After treating cells with warmed trypsin for 5 min at 37 °C to remove membrane-bound tau, we immediately measured tau uptake using flow cytometry or replated the cells at 37 °C for 48 h to detect seeding. Hypothermia reduced tau uptake to below detectable levels (Fig. 5*A*, top) but increased intracellular seeding ∼5-fold relative to 37 °C (Fig. 5*A*, bottom). Heparin abolished this effect by preventing tau binding to HSPGs, confirming that plasma membrane association was required for efficient low-temperature seeding. AD-derived tau seeds immunoprecipitated from human AD brain lysate (Fig. S9*A*) also seeded more effectively at 4 °C than at 37 °C (Fig. S9*B*). To test effects of low temperature on cytoplasmic tau seeding, independent of initial uptake, we incubated cells with fibrils for 4 h at 37 °C to allow normal endocytosis. We then washed the cells and incubated them at 4 °C or 37 °C for 4 h to temporarily disrupt intracellular endocytic processing, with or without subsequent bafilomycin exposure for 4 h at 37 °C, followed by washout, and incubation for 48 h to allow seed replication. When tau seeds were processed through the endocytic pathway at 37 °C, bafilomycin increased seeding, consistent with effects on lysosomal degradation and trafficking. However, following 4 °C exposure, which presumably allowed seed escape into the cytoplasm, bafilomycin had no effect, and seeding was greatly increased overall (Fig. 5*B*). To exclude the possibility that transient hypothermia impaired tau degradation, we examined three different TET-regulated tau stable cell lines that expressed FL-Clover or RD-YFP. Hypothermia did not alter degradation of endogenous full-length or RD tau in Tet-regulated lines (Fig. S8, *A* and *B*), nor did it affect clearance of exogenous fibrils after washing and rewarming (Fig. 5, *C* and *D*).

**Figure 5:**
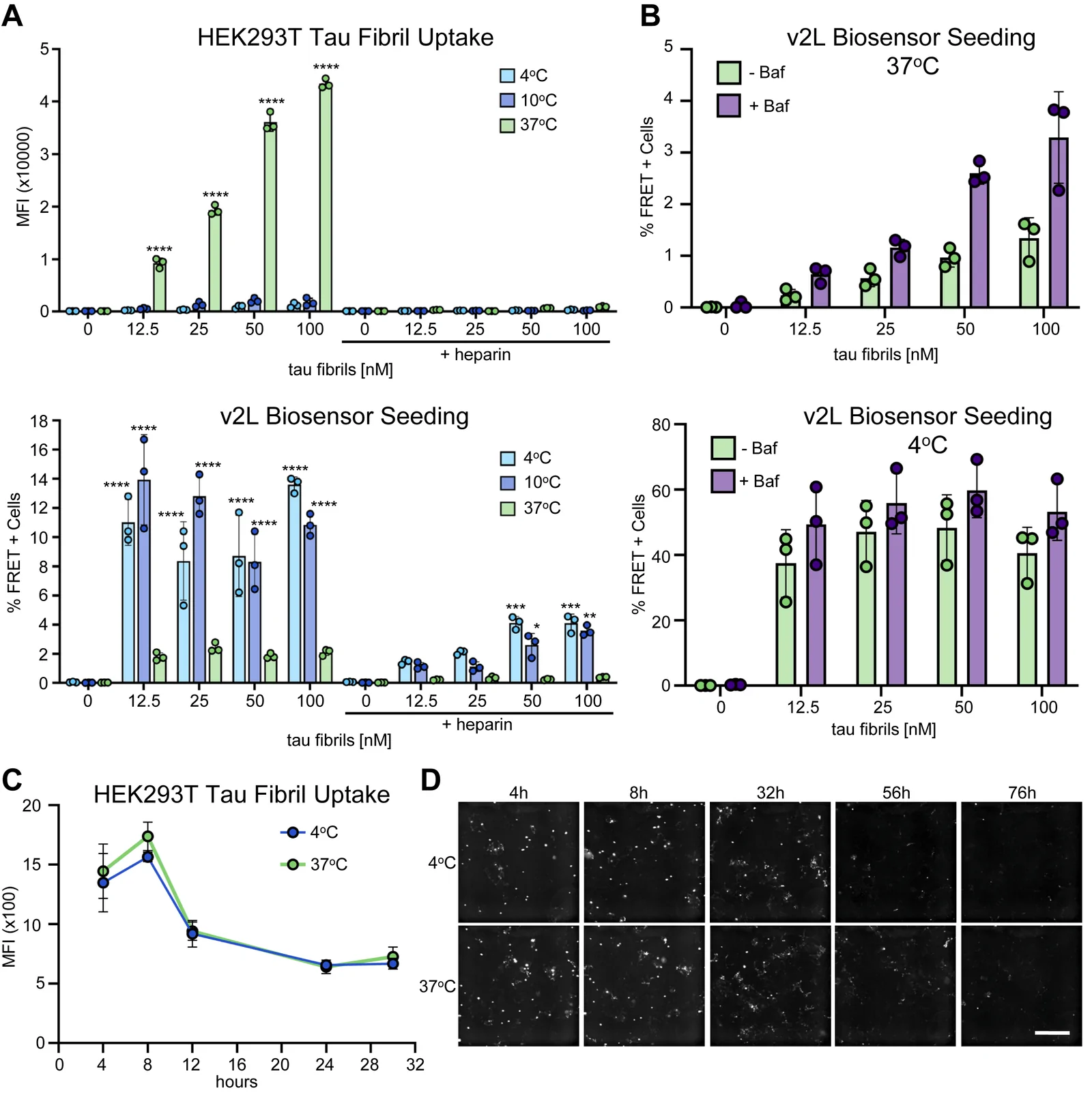
**4 °C or 10 °C incubation reduces tau uptake and increases tau seeding.***A*, HEK293T cells were incubated with the indicated concentrations of sonicated Alexa647-labeled tau fibrils, with or without heparin (200 μg/ml), for 4 h at 4 °C, 10 °C, or 37 °C. Cells were treated with 0.25% trypsin for 5 min at 37 °C and prepared for flow cytometry. *Top*: tau uptake was quantified by flow cytometry as median fluorescence intensity. *Bottom*: tau RD biosensor cells were incubated under the same conditions for 4 h, washed, treated with 0.25% trypsin for 5 min at 37 °C, replated, and incubated for an additional 48 h at 37 °C prior to quantification of intracellular aggregation by FRET flow cytometry. *B*, *top*: tau RD biosensor cells were incubated with tau fibrils for 4 h at 37 °C, washed five times, replated, and incubated at 37 °C for an additional 4 h. Bafilomycin A1 (20 nM) was then added for 4 h, after which cells were trypsinized, replated, and harvested 48 h later. *Bottom*: tau RD biosensor cells were incubated with tau fibrils for 4 h at 37 °C, washed, shifted to 4 °C for 4 h, and then treated with bafilomycin A1 (20 nM) for 4 h prior to trypsinization, replating, and harvest 48 h later. *C*, sonicated Alexa647-labeled tau fibrils were added to HEK293T cells and incubated for 4 h at 37 °C. Cells were harvested at 4 h, after which one plate was shifted to 4 °C and the other maintained at 37 °C for an additional 4 h. Cells were subsequently harvested at 8 h, 24 h, and 30 h. *D*, representative images of HEK293T cells incubated with Alexa647-labeled tau fibrils for 4 h at 37 °C, followed by incubation for an additional 4 h at either 4 °C or 37 °C (8 h total), and then returned to 37 °C for a total of 32 h, 56 h, or 76 h. The scale bar represents 100 μm. All experiments were performed at least twice with similar results. Data represent mean ± SD of technical replicates. Statistical analysis was performed using two-way ANOVA with Dunnett’s multiple-comparisons test compared to 0 nM tau; ∗∗*p* < 0.005, ∗∗∗*p* < 0.0005, ∗∗∗∗*p* < 0.0001. HEK293T, human embryonic kidney 293 T; RD, repeat domain.

## Discussion

By screening for genes that regulated tau fibril uptake, we unexpectedly identified multiple components of the v-ATPase. Across diverse and orthogonal perturbations—genetic, pharmacologic, biochemical, and biophysical—we observed a striking and consistent inverse relationship between tau uptake and intracellular seeding. Surprisingly, in every condition, inhibition of tau uptake correlated with increased seeding. Inhibition of v-ATPase function, expression of dominant-negative Rab5a, cholesterol depletion, or transient hypothermia each markedly reduced tau uptake while simultaneously enhancing seeding efficiency. Efficient endolysosomal trafficking does not appear to be required for tau seeding under the conditions studied here, contrary to our past conclusions, and those of others (9, 13). Instead, efficient endolysosomal trafficking reduces seeding by routing the majority of internalized tau assemblies for lysosomal degradation, indicating that lysosomal trafficking is protective. Tau aggregates that remain at the plasma membrane or within immature endosomal compartments appear far more likely to gain access to the cytoplasm to serve as seeds, whereas those rapidly trafficked to mature lysosomes are neutralized. Together, our data identify membrane transit—not endocytosis *per se*—as a critical gateway for cytoplasmic seeding by pathological tau. We propose a model in which tau binding to cell-surface HSPGs triggers macropinocytosis (9, 10), directing most assemblies toward lysosomal destruction (11), while disruptions in uptake or trafficking—whether through blocked endocytosis or inefficient endosomal maturation—permit a subset of seeds to escape the vesicle and replicate in the cytoplasm (Fig. 6). A limitation of this study is that most experiments used recombinant heparin-induced tau fibrils, which do not fully recapitulate disease-associated tau assemblies.

**Figure 6:**
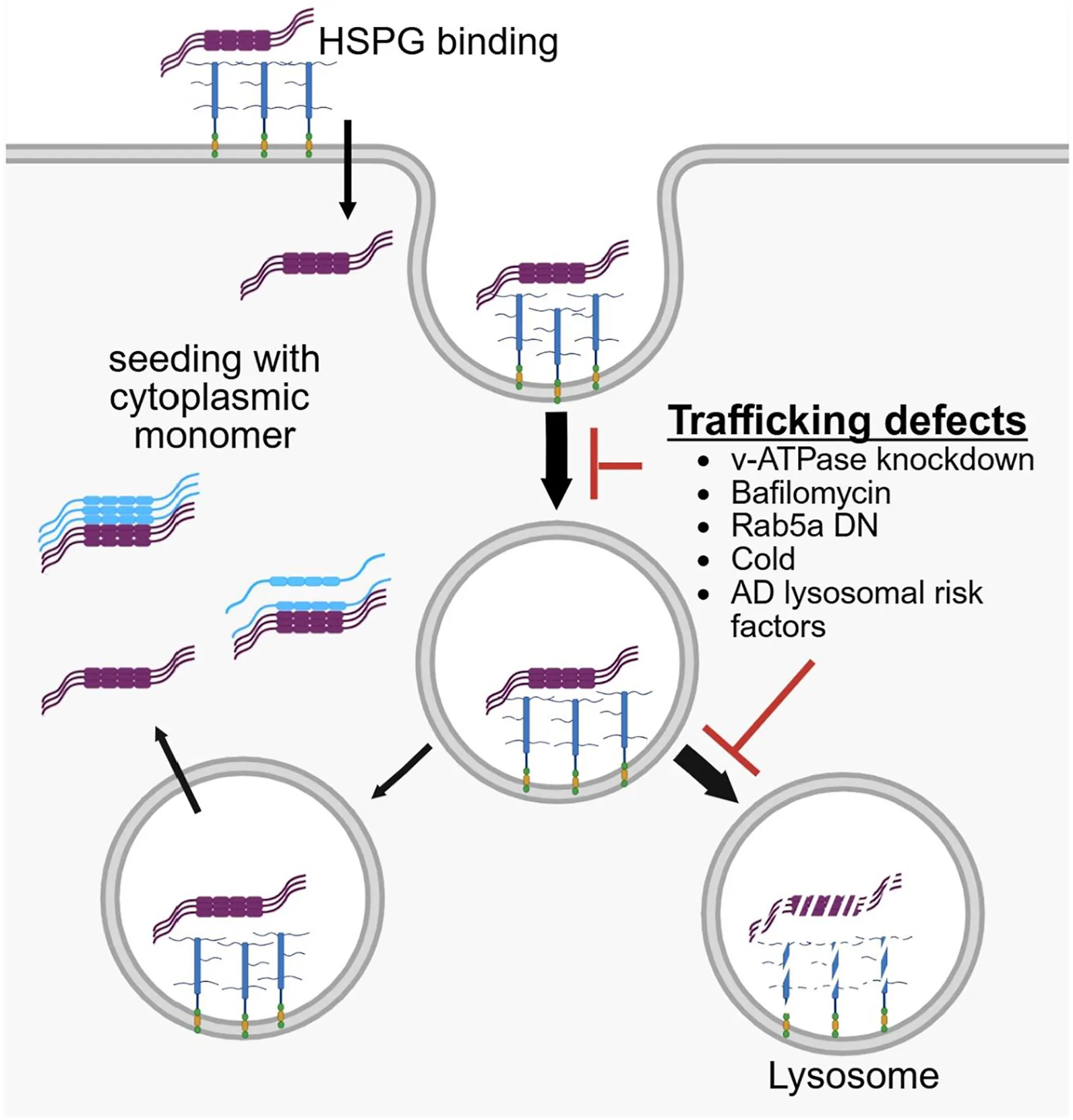
**Model for HSPG-mediated tau uptake and intracellular seeding.** Tau fibrils bind heparan sulfate proteoglycans (HSPGs) on the cell surface, triggering macropinocytosis and trafficking to the endolysosomal system under basal conditions. When endolysosomal function is impaired, this pathway is disrupted, allowing a greater fraction of internalized tau assemblies to access the cytoplasm and seed aggregation. HSPG, heparan sulfate proteoglycan.

### Endosomal processing and tauopathy risk

Vesicle acidification *via* the v-ATPase is required for macropinocytosis and the endocytic uptake of several viruses (32, 33, 34), and a whole-genome CRISPR screen in iPSC neurons has previously identified v-ATPase components critical for tau fibril uptake (35). Importantly, numerous AD risk genes have been linked to endocytosis, including *BIN1*, *PICALM*, *CD2AP*, *EPHA1*, and *SORL1* (36, 37), and whole-exome sequencing in early-onset AD has identified multiple risk-associated genes involved in endolysosomal transport (38). Consistent with our findings, reduction of BIN1 increases tau seeding in cell models (39), and endocytic pathway abnormalities—including Rab5-positive early endosome enlargement and lysosomal defects—precede overt amyloid pathology in the brain. Indeed, others have suggested that disrupted trafficking within the endocytic pathway is linked to AD pathogenesis, although mechanisms have not been determined (22, 40). Disruption of v-ATPase activity has also been previously implicated in neurodegenerative diseases (41). Even before amyloid beta deposits appear in the brain, endocytic pathway abnormalities such as Rab5-positive early endosome enlargement, phagocytic vacuole accumulation and defects in lysosomes have been described (38, 42, 43). Furthermore, cholesterol depletion, in association with vesicle trafficking defects, has previously been reported to promote cytosolic entry of tau aggregates (29). Separate from endolysosomal degradation, which is also a potential regulator of seeding efficiency, our findings underscore the role of vesicle trafficking in seeding from the outside to the inside of the cell. Specifically, if tau seeds enter the vesicular compartment but are not trafficked efficiently, they may access the cytoplasm *via* transient membrane permeabilization (13). Rather than reflecting impaired degradation alone, defects in endolysosomal trafficking may directly enhance tau seeding by facilitating access of seeds to the cytoplasm. These findings caution that manipulation of endocytic or autophagic pathways for therapy may unintentionally increase seeding.

### Endocytosis and seeding by other amyloid proteins

This study did not evaluate the role of endolysosomal processing in the seeding of other amyloid-forming proteins that have been proposed to mediate transneuronal spread of pathology. However, several observations suggest that the principles identified here may extend beyond tau. For example, α-synuclein fibrils bind HSPGs and can enter cells through endocytic pathways (9, 44, 45), although the molecular determinants of uptake are less well defined than for tau. Our findings suggest that efficient endolysosomal trafficking may serve a protective role by directing internalized protein aggregates toward degradation, thereby limiting access to the cytoplasm. Whether inhibition of endocytosis or disruption of endosomal maturation similarly enhances cytoplasmic entry and seeding of α-synuclein or other amyloid proteins remains unknown. Determining whether membrane transit occurs independently of productive endocytosis represents an important direction for future investigation and may reveal a common mechanism governing the seeding in diverse protein aggregation disorders.

### Tau uptake and seeding are separable

We previously identified macropinocytosis as the primary route of tau uptake and seeding after binding of tau assemblies to cell-surface HSPGs (9). While our data indicated that tau must efficiently bind surface HSPGs for intracellular seeding, macropinocytosis itself is not required. Macropinocytosis routes most tau seeds into a lysosomal degradation pathway (11), while the small percentage of tau destined to replicate in the cytoplasm must escape the vesicular compartment or cross the plasma membrane to contact endogenous tau. The dependence of seeding efficiency on vesicle trafficking has important implications for therapeutic strategies aimed at modulating endocytosis or lysosomal function, as these may increase cytoplasmic seeding. Our findings underscore the need to distinguish pathways that promote aggregate clearance from those that might paradoxically promote pathological tau seed amplification.

## Experimental procedures

### Cell culture

HEK293T/17 cells (ATCC CRL-11268) were cultured in Dulbecco's modified Eagle's medium supplemented with 10% FBS and GlutaMax, in the absence of penicillin-streptomycin, and grown in a humidified atmosphere of 5% CO_2_ at 37 °C. Tet-OFF-Tau-RD cells (TO-1) and Tet-OFF-Tau-RD constitutively seeding cells (OFF11DS11) were described previously (46). Tet-OFF-full-length-Tau FRET biosensor cells were generated by transducing HEK293T cells with two lentivirus constructs (FM5 plasmids) encoding FL P301S tau (2N4R) tagged either mClover3 or mCerulean3 and whose expression is regulated by tetracycline-controlled transcriptional activation. Cells expressing tau were selected and sorted in a BD FACS Aria II system (BD Biosciences). The FACS sorted populations were replated in a 96 well plate to obtain a monoclonal stable population. Cells were grown approximately 3 weeks, with tau expression confirmed by Western blot, and biosensor activity *via* FRET flow-cytometry. All cell lines were routinely checked for *mycoplasma* contamination using the PCR-based VenorGeM detection kit and found to be negative.

### Differentiation and culturing of human iPSC-derived cortical neurons

We utilized the integrated, inducible, and isogenic neurogenin-2 (Ngn2) iPSC line (i^3^N). It was previously shown that expression of the transcription factor Ngn2 induces rapid differentiation of iPSCs into glutamatergic neurons (47). This iPSC line harbors a doxycycline-inducible mouse Ngn2 transgene at an adeno-associated virus integration site 1 safe-harbor locus, allowing for a simplified differentiation protocol (48). iPSCs were dissociated with accutase (Sigma-Aldrich, A6964) and plated onto basement membrane extract-coated plates (R&D, 3434-001-02). Ngn2 expression was induced with 2 μM doxycycline hyclate (Sigma-Aldrich, D9891) in KSR media alone with 10 μM SB431442 (R&D, 1614), 2 μM XAV939 (Stemgent, 04–0046) and 100 nM LDN-193189 (Stemgent, 04-0074), with doxycycline hyclate was maintained in all media going forward. On day 2, cells were fed with a 1:1 ratio of KSR media + SB/XAV/LDN and N2-supplmented neural induction media with 2 μg/ml puromycin (Life Technologies, A1113803). On day 3, cells were fed with N2-supplemented neural induction media. On day 4, cells were dissociated with accutase (Sigma-Aldrich, A6964) and plated onto PDL-(Sigma-Aldrich, P1149) and laminin-(Life Technologies, 23017-015) coated tissue culture plates. Cells were subsequently maintained with neurobasal media (Life Technologies, 21103049) supplemented with NeuroCult SM1 (StemCell Technologies, 05711) and 10 ng/ml brain-derived neurotrophic factor (R&D, 248-BD-005/CF). See Fig. S4.

### Protein preparation, fibrillization, and labeling of recombinant tau

Full length 2N4R tau was subcloned into pRK172. Protein was prepared from Rosetta (DE3) pLacI competent cells using a modified protocol described previously (Goedert and Jakes). *E. coli* were grown in Terrific Broth to an optical density of 1.45 at 600 nm. IPTG at 1 mM was added, and cells were grown for 2 hs after which bacteria were harvested and flash frozen. The frozen pellet was resuspended in 1 X Brinkley reassembly buffer 80 (BRB-80) buffer [400 mM PIPES pH 6.8, 5 mM MgSO4, 5 mM ethylene glycol tetraacetic acid, and 0.1% β-mercaptoethanol] with 1 mM phenylmethylsulfonyl fluoride, 5U/ml DNAse and 1 U/ml RNAse. A PandaPlus 2000 French press lysed the cells. The supernatant was boiled for 10 min, centrifuged at 15,000*g* for 20 min and filtered through a 0.22 μm unit. The filtrate was loaded onto a AKTA FPLC with a HiTrap SP HP 5 ml column. Gradient elution was performed with 1X BRB-80 buffer and 1 M NaCl in BRB-80 buffer. Column fractions were run on an SDS-PAGE to determine fractions with tau. Tau fractions were pooled and guanidinium chloride was added to a total of 4 M. Dialysis was performed with 4 L of 1X tau buffer [10 mM Hepes pH 7.4, 100 mM NaCl] overnight with one buffer exchange. Tau was then fibrillized with 8uM tau monomer, 8 μM heparin and 10 mM DTT in 1X tau buffer for 3 days at 37 °C without shaking. To fluorescently label fibrils, 200 μl of 8 μM tau fibrils were incubated with 0.025 mg of AlexaFluor 647 succinimidyl ester dye for 1 h at RT. After quenching for 1 h with 100 mM glycine the fibrils were dialyzed into PBS overnight with two exchanges of buffer. Tau fibrils were vortexed, briefly spun down, sonicated for 30 s at amplitude 65 in a cold water bath, vortexed and briefly spun down immediately prior to all uptake and seeding assays.

### Genome-wide CRISPR screen for tau uptake

#### Library production

The human GeCKO lentiviral sgRNA library v2 (LentiCRISPR) was obtained from Addgene and plasmids were amplified according to the instructions. Lentivirus for Library A and Library B was prepared separately in HEK293T cells in 10 cm dishes transfected using 30 μl Fugene HD with 5 μg Gecko library, 3 μg psPAX2 and 2 μg pMD2.G in 900 μl Opti-MEM. Media was exchanged after 8 h. At 24 and 48 h after media exchange, the media was harvested and pooled. The lentivirus-containing medium was filtered through a 0.45 μm filter and stored at −80 °C. The libraries were titered on HEK293T by testing different volumes for puromycin (Thermo Fisher Scientific) resistance and quantitating cell numbers by Cell-Titer-Glo (Promega).

#### Transduction of HEK293T with the GeCKO lentivirus library

Biological replicates were used for each library. HEK293T were plated onto multiple 12-well plates and transduced at a MOI of 0.3 with 8 μg/ml polybrene (Millipore). The following day, cells were harvested and plated into a T300 flask. A total of 48 h later cells were harvested, pooled, and 1 μg/ml puromycin (Thermo Fisher Scientific) was added to the cells. Cells were expanded and kept in puromycin for 19 days. The day before sorting, cells were split for the tau uptake assay to be collected by FACS and also for use as an unsorted control.

#### Tau uptake and cell sorting

Alexa-647-labeled recombinant tau fibrils were sonicated for 30 s at amplitude 6.5 and diluted into media to a final concentration of 25 nM. . Tau was added to cells transduced with the sgRNA library for 4 h at 37 °C. 0.25% trypsin was added for 5 min and cells were resuspended. >2.4 × 10^8^ cells were sorted 2 separate times on a FACS Aria II SORP, once quickly with a fast, non-stringent Alexa-647 gate, pooled and resorted with a slower, more stringent Alexa-647 gate. As cells were being sorted, a 500× overrepresentation of cells with sgRNAs was harvested as the nonsorted control. Each library, A and B, was collected in a separate week.

#### Genomic DNA extraction and sequencing

All gDNA extraction was performed with a cell lysis buffer and a phenol:chloroform extraction as described (49). The sequencing was performed by two rounds of PCR as described (49).

#### Bioinformatics analysis

Samples were sequenced on Illumina NextSeq 500 with read configuration as 76 bp, single end. The fastq files were subjected to quality check using fastqc (version 0.11.2, http://www.bioinformatics.babraham.ac.uk/projects/fastqc) and fastq_screen (version 0.4.4, http://www.bioinformatics.babraham.ac.uk/projects/fastq_screen), and adapters trimmed using an in-house script. The reference sgRNA sequences for human GeCKO v2.0 (A and B) were downloaded from addgene (https://www.addgene.org/pooled-library/). The trimmed fastq files were mapped to reference sgRNA library with mismatch option as 0 using MAGeCK (50). Furthermore, read counts for each sgRNA were generated, and median normalization was performed to adjust for library sizes. Positively and negatively selected sgRNA and genes were identified using the default parameters of MAGeCK. Pathway enrichment analysis was performed using ingenuity pathway analysis tool (IPA, http://www.ingenuity.com). The complete dataset is provided in Table S1.

### Lentivirus production

Two separate human guide RNA sequences for RNAseK and ATP6AP2 along with two non-targeting gRNA sequences and NDST1 were selected from the GeCKO version 2 library.

**Table:** 

Gene id	sgRNA sequence
RNAseK-1	AACGTCCTCAATCAACACAG
RNAseK-2	CCATTCCGCTGTGTTGATTG
ATP6AP2-1	TTTCGGAAAACAACAGACCC
ATP6AP2-2	GTAACCTGTTTCATCGTCCT
NDST1	CGTGCACGTGCAGCTGTACG
NT0460	GGCTTACGTGGGGGGCAAAA
NT0849	GGGAGGTGGCTTTAGGTTTT

DNA oligonucleotides were synthesized by IDT DNA and cloned into the lentiCRISPRv2 vector (17). Lentiviral plasmids for expression of all dominant negative proteins (Rab5aS34N, Rab7aN125I, Cdc42T17N, CAV1Y14F, Eps15 delta95/295, DNM2 K44A, and Arf6T27N) along with TagRFP were provided by the Schoggins lab (51). Lentiviruses were created as described (10). Lentiviruses were titered by serial dilution and either puromycin selection along with Cell-Titer-Glo (CRISPR constructs) or TagRFP expression along with flow cytometry (dominant negative protein constructs).

### Inference of CRISPR edits analysis of CRISPR KOs

HEK293T cells were transduced with lentivirus containing sgRNAs at an MOI of 5. Cells were selected in puromycin for 8 days. Genomic DNA was isolated from the cells using a DNeasy blood and tissue kit (Qiagen). Regions around the sgRNA cut site were amplified by PCR, and bands were gel extracted. DNA from the PCR reaction was sent for Sanger sequencing. Results were uploaded to the Synthego website (https://ice.synthego.com/#/) for analysis.

### Tau fibril uptake assay for HEK293T and iPSC neurons

HEK293T cells were plated at 20,000 cells/well in a 96-well plate. iPSC neurons were initially plated at 10,000 cells/well in a glass-like 96-well plate. Tau fibrils labeled with Alexa 647 were sonicated for 30 s at 65 amp. 25 nM of these sonicated tau fibrils were added to the wells. For the uptake timecourse (Fig. 1) 250 nM transferrin-Alexa647 (Thermo Fisher Scientific T23366), 1.43uM dextran-TexasRed (Thermo Fisher Scientific D1830), or 5uM TAT (AnaSpec AS60023) labeled with Alexa647 were added to the wells. As indicated, some wells also had heparin (amsbio HEP0001-100) added. Plates were incubated at 37 °C for varying timepoints. 0.25% trypsin was added for 5 min to digest tau fibrils on the cell surface. Harvested cells were then fixed with 2% paraformaldehyde and prepared in flow buffer.

### Tau seeding assay

v2L tau biosensor cells were previously reported and characterized (16). They are derived from HEK293T cells and express tau RD fused to cerulean or clover fluorescent proteins. For seeding assays, they were plated at 15,000 cells/well in a 96-well plate. The next day, tau fibrils were sonicated for 30 s at 65amp. A total of 50 nM of sonicated fibrils were added to the cells and incubated at 37 °C for 48 h. Cells were detached with trypsin, fixed with 2% paraformaldehyde, and prepared for flow cytometry.

### Bafilomycin A treatment

Bafilomycin A1 (Sigma-Aldrich Bd) was used in several experimental designs as describe below for each figure panel:

Figure 2, *A* and *B* (HEK293T cells-uptake and seeding with varying bafilomycin concentrations): HEK293T cells were pretreated with bafilomycin for 0.5 h after which bafilomycin and sonicated 25 nM tau fibrils were added for 4 h. For the uptake assay (Fig. 2*A*), tau fibrils were labeled with Alexa647. For the seeding assay (Fig. 2*B*), unlabeled tau fibrils were used, and following the 4 h incubation cells were washed twice with media and incubated at 37 °C for 48 h prior to harvesting. In both experiments, 200 μg/mL heparin was included during the 4 h tau fibril incubation as a control. At the end of each experiment cells were harvested using warmed 0.25% trypsin for 5 min and prepared for flow cytometry.

Figure 2*C* (iPSC neurons – uptake with bafilomycin): iPSC neurons were incubated with Alexa 647-labeled, sonicated tau fibrils and bafilomycin at varying concentrations, or with 200 μg/mL heparin, at various concentrations for 4 h. Cells were then washed three times with warmed media and returned to the 37 °C incubator for 48 h. Cells were subsequently harvested using warmed 0.25% trypsin for 5 min and prepared for flow cytometry.

Figure 2*D* (iPSC neurons – seeding with bafilomycin): iPSC neurons converted to biosensor cells by transduction with lentivirus to express tau RD (P301S) Clover and Ruby. Four days after transduction 20 nM sonicated tau fibrils and bafilomycin were added simultaneously, with 200 mg/ml heparin included as a control. Following a 4 h incubation, cells were carefully washed to remove all bafilomycin and incubated for 48 h to allow for seeding. FRET analysis was performed by confocal microscopy with image analysis as described in the FRET Analysis of iPSC Neurons section below.

Figure 2*E* (HEK293T biosensor cells – imaging): HEK293T biosensor cells were not pretreated with bafilomycin. Instead bafilomycin (or 200 μg/mL heparin as a control) was added simultaneously with 25 nM sonicated tau fibirls. After the 4 h incubation period, cells were harvested with warmed 0.25% trypsin for 5 min, replated, and incubated at 37 °C for 48 h prior to imaging on a confocal microscope. After imaging, cells were incubated with warmed 0.25% trypsin for 5 min, harvested and prepared for flow cytometry. Analysis of these cells are shown in Figure 2*F*.

### Testing individual CRISPR KOs for tau uptake and seeding

HEK293T cells (for uptake assays) or v2L cells (for seeding assays) were plated at 2 × 10^5^ cells/well in a 24 well dish and subsequently split for maintenance. Two days later, cells were split and cultured with puromycin. After 5 days cells were split at 15,000 cells/well, and biosensor cells were split at 20,000 cells/well in 96 well plates. The next day uptake assays or biosensor seeding assays were performed as described previously.

### Tau fibril seeding assay in iPSC neurons

iPSC neurons were initially plated at 10,000 cells/well on a clear 96-well plate. Cells were maintained for a month in media with BDNF and doxycycline. Laminin (Sigma-Aldrich) at 1 mg/ml was added a few days before the experiment. Lentiviruses expressing tauRD-Clover and tauRD-Ruby under a ubiquitin promoter were added to cells at an MOI of 3 and allowed to express for 4 days. Bafilomycin and 20 nM tau fibrils were added to cells for 4 h. Cells were then washed and placed back in the incubator for 3 days. Cells were fixed, kept in PBS and analyzed on an IN Cell Analyzer 6000 (Molecular Devices) collecting 9 data points for each well with lasers tuned for GFP, dsRed, and FRET with a 20× objective using MetaXpress software.

### FRET analysis of iPSC neurons

#### Background correction

To eliminate image noise in subsequent analysis, all images underwent background correction as follows: a Gaussian filter with a sigma value of 100 was applied to the image and this filtered version was subtracted from the original image.

#### Cell segmentation

To segment cells within an image, we first calculated the image gradient of the donor channel, using the Sobel method, and normalized this output by the gradient magnitude. We then subtracted the resulting normalized image gradient from the original image to enhance cell edges in the image. To remove remaining image noise, we applied a Gaussian filter with sigma value of 5. Last, we calculated the Rosin Threshold from the filtered image and used this threshold value to segment cell boundaries.

#### Calculation of FRET signal

To correct for bleed-through from other imaging channels (donor and acceptor), we calculated a linear fit (using the polyfit function) for fluorescence intensity between the channel of interest (FRET Channel) and either the donor or acceptor channel. The slopes from these calculations were then used in the following function to calculate the corrected FRET image.

The average FRET intensity for each image was then calculated from the corrected image for all values within the cell segmentation.

### Dominant negative protein expression for uptake and tau seeding experiments

HEK293T or biosensor cells were plated at 3.4 × 10^4^ cells/well in a 12 well dish and transduced with lentivirus at an MOI of 3, and split to maintain proper density. Two days later, cells were split into a 96 well plate in triplicate. The next day uptake assays or biosensor seeding assays were performed as described previously.

### Cold treatment of cells

HEK293T cells were plated as for the uptake or seeding assays on 96-well plates. The next day an Eppendorf Thermomixer was fitted with a plate adapter and prechilled to 10 °C. Samples of tau were prepared in round bottom 96-well plates that were either put on ice (for the 4 °C and 10 °C samples) or left at RT (for the 37 °C plate) while tau fibrils were added. Plates were then incubated for 4 h either at 37 °C, in a 4 °C cold room, or on a Thermomixer set at 10 °C (not mixing). Media was then removed, warmed 0.25% trypsin added, and cells were placed in the 37 °C incubator for 5 min. Warmed media was then added and the cells were gently resuspended. Plates for uptake were then prepared for flow cytometry to quantitate the cells with fluorescence. Cells for the seeding assay were replated and incubated for 2 days at 37 °C before flow cytometry and FRET analysis.

### Tau immunoprecipitation from AD brain lysate

Human brain tissue was obtained from the brain bank of the Alzheimer’s Disease Center of UT Southwestern. The brain was from an 83-year-old female with a 20-years history of Alzheimer’s-type dementia. Neuropathology showed high-level Alzheimer disease neuropathologic change, with Braak stage VI (neocortical stage) neurofibrillary tangle pathology. There was no neuropathologic evidence of concomitant synucleinopathy, TDP-43 pathology, or ischemic lesions. The *postmortem* interval (time between death and autopsy) was 8 h. Brain weight was 775 g. Frozen frontal cortex tissue from this brain was homogenized in 10% w/vol of 1 x TBS with protease inhibitor cocktail (Roche) at 4 °C using an electric homogenizer with disposable probes (Power Gen 125 and Hard Tissue Omni Tip Plastic Homogenizing Probe) followed by intermittent 4 °C water bath sonication for 15 min at 65 amplitude (30 s on, 60 s off). Homogenates were centrifuged at 21,000 × *g* for 15 min at 4 °C to remove cellular debris. Protein concentration was determined by BCA assay.

For the Western blot with brain lysate, each lane was loaded with the equivalent of 2 μg of total protein in a 4 to 12% bis-tris SDS-PAGE. After transferring to PVDF membrane, the blot was blocked for 1 h with 5% milk in 0.1% TBS-T at RT. The membrane was incubated overnight at 4 °C in a 1:2000 solution of polyclonal rabbit anti-human tau IgG (Dako A0024). The blot was washed and incubated for 2 h at RT in a 1:8000 solution of HRP-Linked Donkey anti-Rabbit IgG and then developed (ECL Prime Western Detection kit, Amersham). A lane loaded with recombinant tau monomer was added for verification of the anti-tau antibody. Detection was done on a Syngene G:Box.

For the immunoprecipitations, 250 μl of Protein A Dynabeads were washed in 1 ml of 0.02% PBS-T and brought up in 50 μg of Tau B antibody (epitope KIGSTENL). After 1 h at RT of rotating end-over-end beads were washed with 0.02% PBS-T and resuspended in 160 μl of AD brain lysate (2.073 mg/ml) with 440 μl of PBS, and rotated overnight at 4 °C. They were then washed twice with IgG elution buffer (Thermo Fisher Scientific). Subsequently, Tris buffer pH 8.5 was added. For the seeding assay biosensor cells were plated in a 96-well dish. Either tau-immunoprecipitated AD lysate or recombinant tau fibrils were added to the cells either on ice or at RT. Cells were then moved to 4 °C or 37 °C respectively for 4 hs. Cells were then treated with warmed trypsin for 5 min at 37 °C, replated, and incubated at 37 °C for 2 days. Cells were then harvested and counted on a flow cytometer for FRET analysis.

### MBCD treatment to deplete cholesterol within cells

To check for toxicity, HEK293T cells were plated at 25,000 cells/well in a 96 well plate the day before the experiment. MBCD (Sigma-Aldrich M7439) was prepared fresh the day of the experiment by making 500 mM in 25 mM Hepes pH7.4 and filtering. Different concentrations of MBCD were prepared in Dulbecco's modified Eagle's medium without fetal bovine serum. Media was removed from the cells and 100 uL of the MBCD solution was added to the well. Cells were incubated at 37 °C for 4.5 h. Cell-Titer-Glo (Promega) was used to determine cell viability. Numbers of cells within a well was calculated based on a standard curve of known amounts of cells. For the experiment with tau uptake and tau seeding HEK293T cells (27,000 cells/well) and biosensor cells (25,000 cells/well) were plated the day before the experiment. MBCD was prepared as before and added to wells. Plates were incubated at 37 °C for 0.5 h. Media was removed and replaced with tau fibrils and MBCD. Plates were then incubated at 37 °C for 4 h. HEK293T cells for uptake analysis were harvested while biosensor cells for seeding analysis were trypsinized, replated, and incubated at 37 °C for 2 days before harvesting for flow cytometry to determine MFI (uptake) and FRET (seeding).

## Data availability

All data supporting the findings of this study are contained within the article and its Supporting Information.

## Supporting information

This article contains supporting information.

## Conflict of interest

The authors declare that they have no conflicts of interest with the contents of this article.
