# Supplementary material for: Endolysosomal inhibition uncouples tau uptake from intracellular seeding
Source: J Biol Chem. 2026 Aug 27;302(10):113498. doi: 10.1016/j.jbc.2026.113498 (PMC13671035; doi:10.1016/j.jbc.2026.113498)

Figure S7

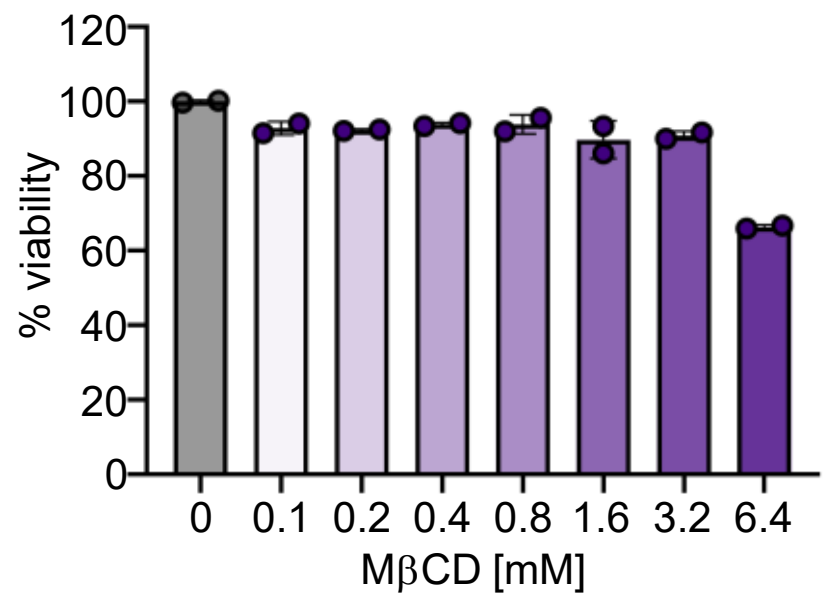

Supplement: Supplementary file 1 [file mmc10.pdf]
