# Supplementary material for: Endolysosomal inhibition uncouples tau uptake from intracellular seeding
Source: J Biol Chem. 2026 Aug 27;302(10):113498. doi: 10.1016/j.jbc.2026.113498 (PMC13671035; doi:10.1016/j.jbc.2026.113498)
Supplement: Supplementary file 2 [file mmc11.pdf]

Figure S8

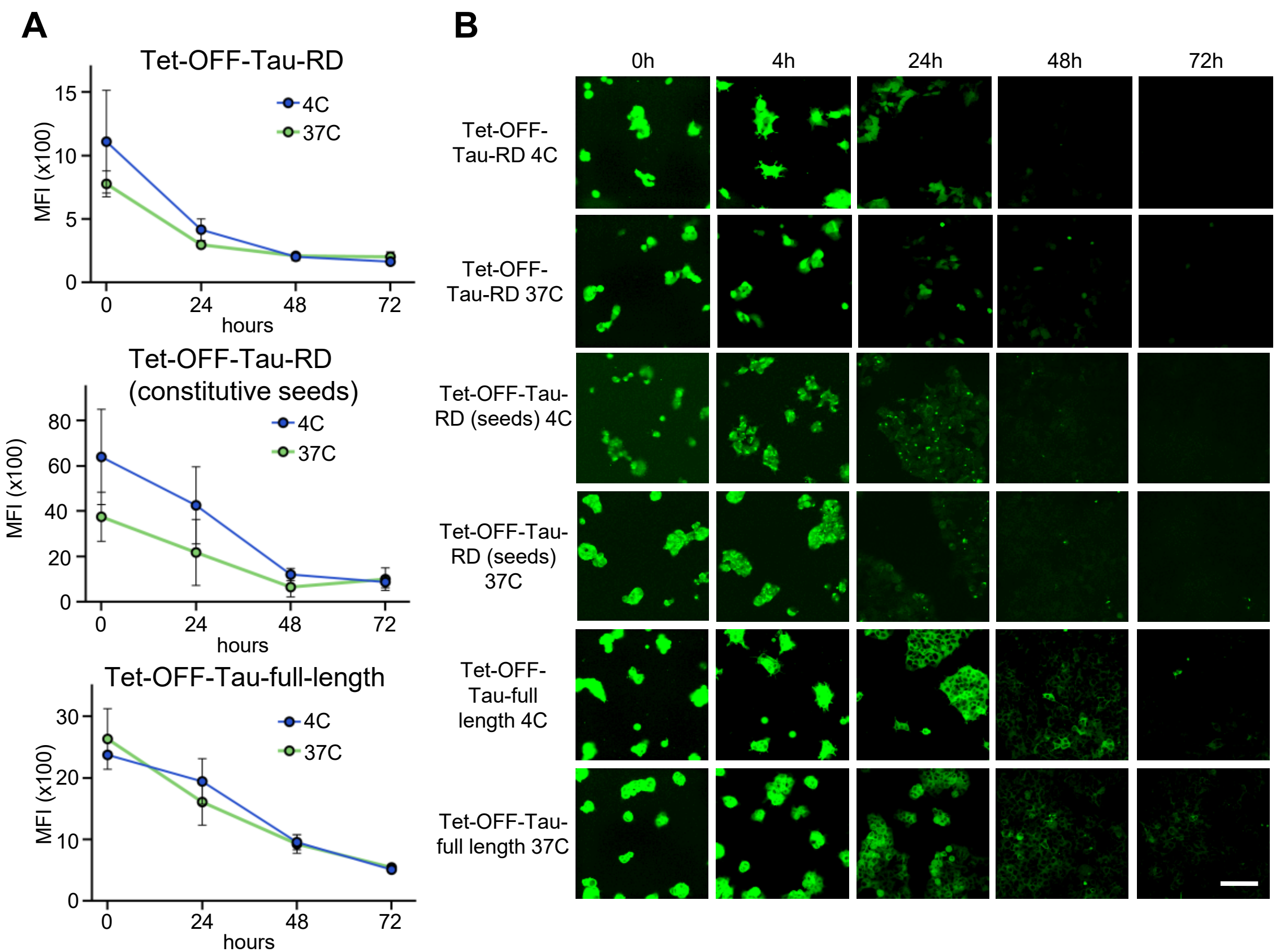
A
Tet-OFF-Tau-RD
MFI (x100)
15
10
5
0
0
24
48
72
hours
4C
37C
Tet-OFF-Tau-RD
(constitutive seeds)
MFI (x100)
80
60
40
20
0
0
24
48
72
hours
4C
37C
Tet-OFF-Tau-full-length
MFI (x100)
30
20
10
0
0
24
48
72
hours
4C
37C
B
0h
4h
24h
48h
72h
Tet-OFF-Tau-RD 4C
Tet-OFF-Tau-RD 37C
Tet-OFF-Tau-RD (seeds) 4C
Tet-OFF-Tau-RD (seeds) 37C
Tet-OFF-Tau-full length 4C
Tet-OFF-Tau-full length 37C
