# Supplementary material for: Endolysosomal inhibition uncouples tau uptake from intracellular seeding
Source: J Biol Chem. 2026 Aug 27;302(10):113498. doi: 10.1016/j.jbc.2026.113498 (PMC13671035; doi:10.1016/j.jbc.2026.113498)
Supplement: Supplementary file 3 [file mmc12.pdf]

Figure S9

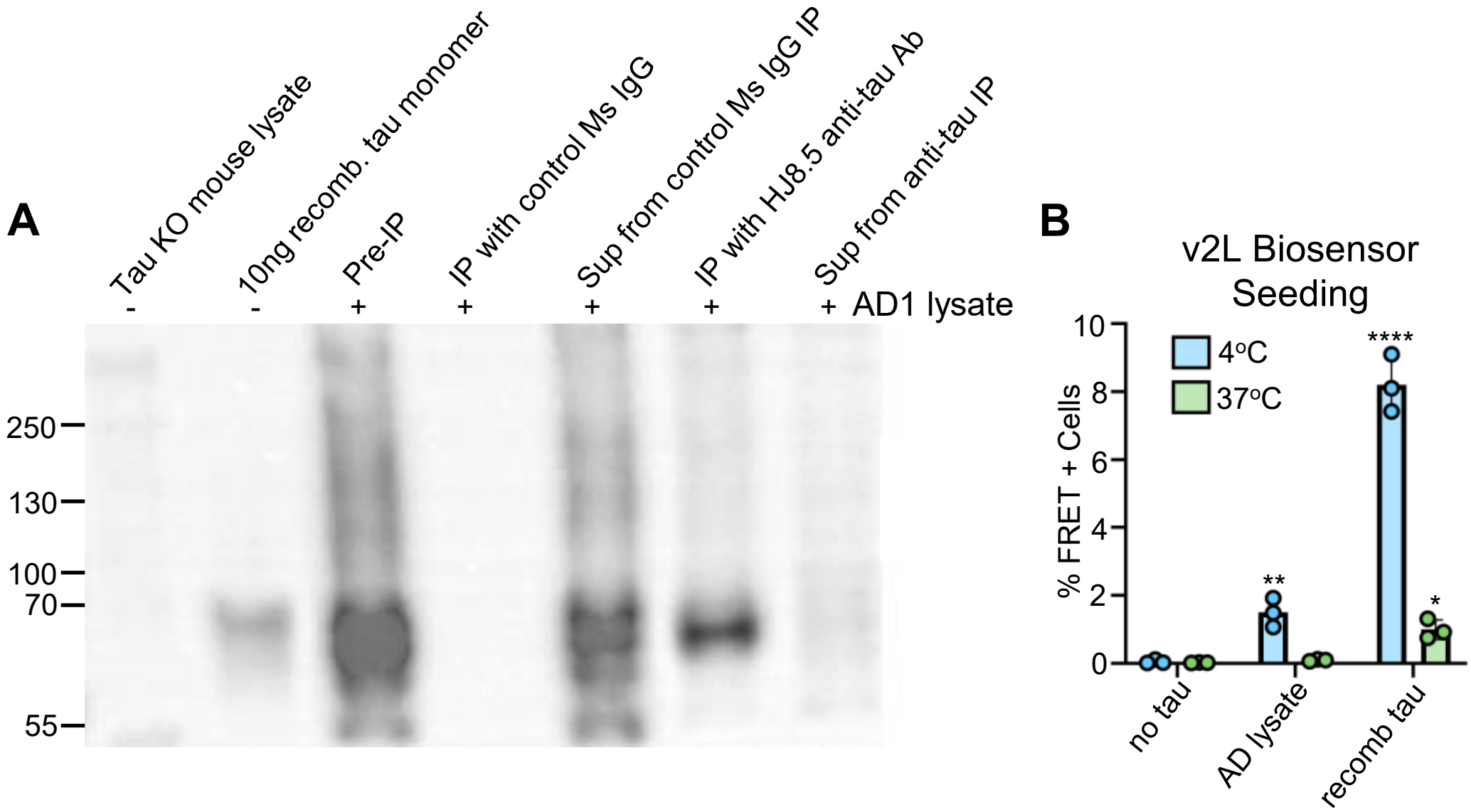
A
Tau KO mouse lysate
10ng recomb. tau monomer
Pre-IP
IP with control Ms IgG
Sup from control Ms IgG IP
IP with HJ8.5 anti-tau Ab
Sup from anti-tau IP
- - + + + + + AD1 lysate
250
130
100
70
55
B
v2L Biosensor Seeding
% FRET + Cells
4oC
37oC
**
****
*
no tau
AD lysate
recomb tau
