# Supplementary material for: Endolysosomal inhibition uncouples tau uptake from intracellular seeding
Source: J Biol Chem. 2026 Aug 27;302(10):113498. doi: 10.1016/j.jbc.2026.113498 (PMC13671035; doi:10.1016/j.jbc.2026.113498)
Supplement: Supplementary file 4 [file mmc4.pdf]

Figure S1

A

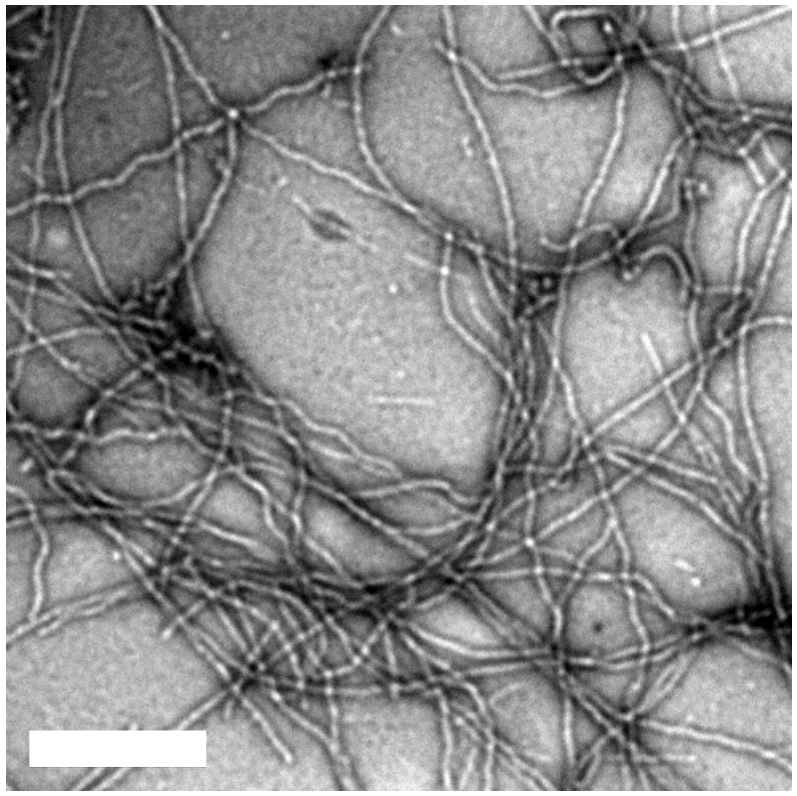

B

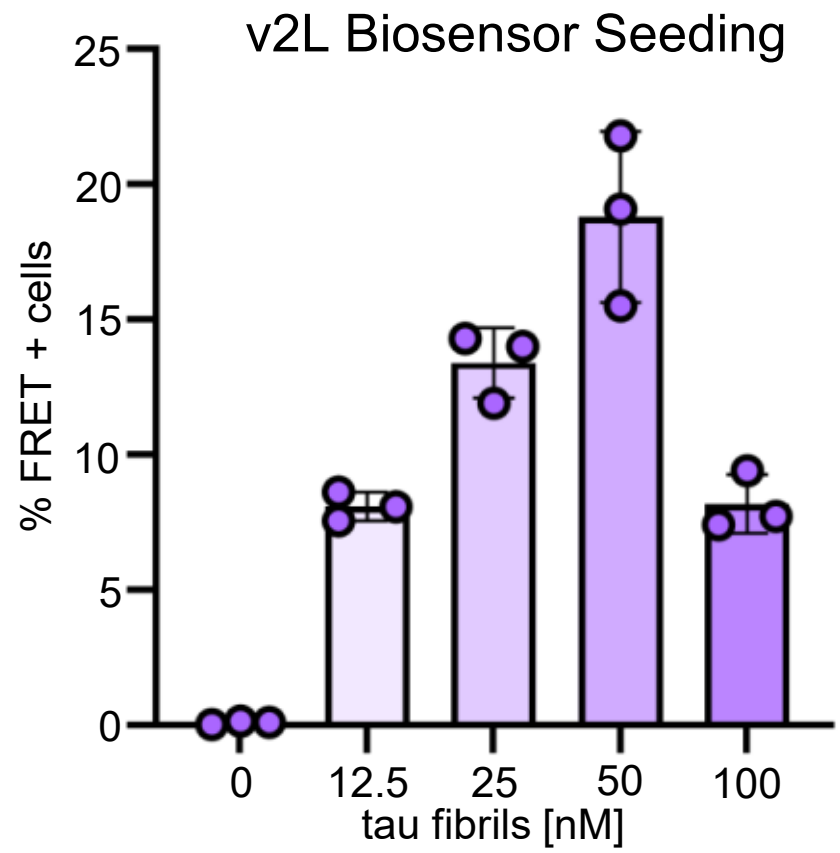
v2L Biosensor Seeding
% FRET + cells
25
20
15
10
5
0
0
12.5
25
50
100
tau fibrils [nM]
