# Supplementary material for: Endolysosomal inhibition uncouples tau uptake from intracellular seeding
Source: J Biol Chem. 2026 Aug 27;302(10):113498. doi: 10.1016/j.jbc.2026.113498 (PMC13671035; doi:10.1016/j.jbc.2026.113498)
Supplement: Supplementary file 5 [file mmc5.pdf]

Figure S2

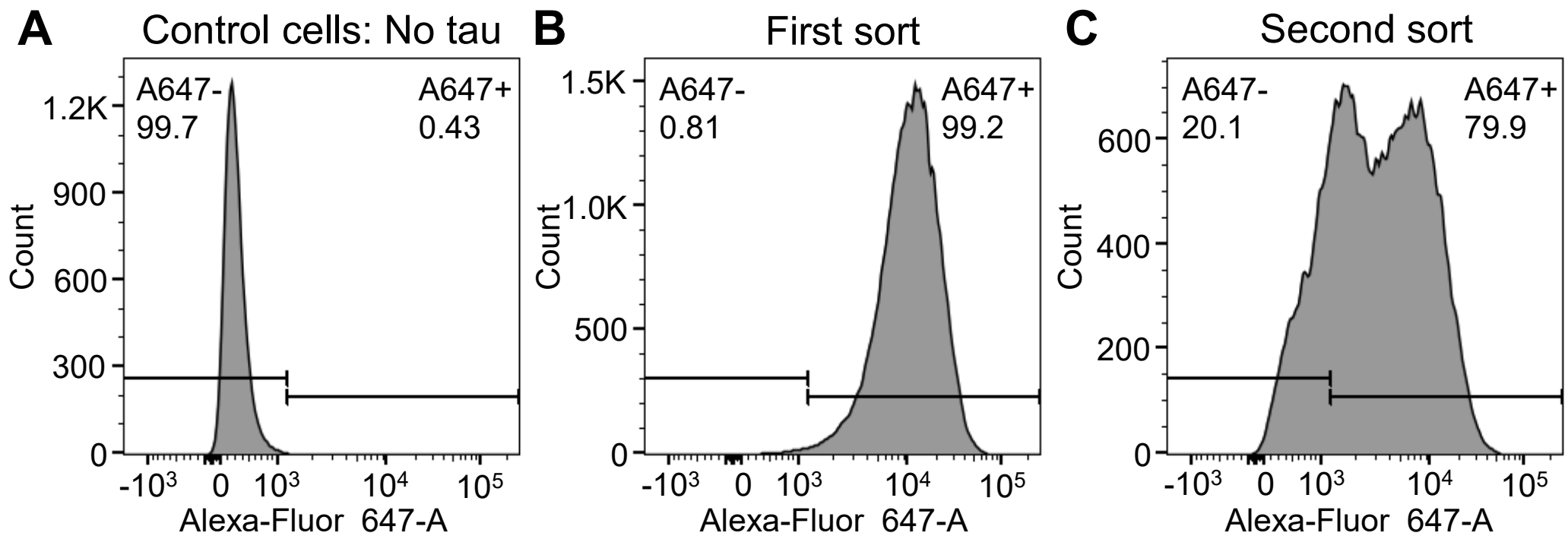
A
Control cells: No tau
A647-
99.7
A647+
0.43
1.2K
900
600
300
0
Count
-10³
0
10³
10⁴
10⁵
Alexa-Fluor 647-A
B
First sort
A647-
0.81
A647+
99.2
1.5K
1.0K
500
0
Count
-10³
0
10³
10⁴
10⁵
Alexa-Fluor 647-A
C
Second sort
A647-
20.1
A647+
79.9
600
400
200
0
Count
-10³
0
10³
10⁴
10⁵
Alexa-Fluor 647-A
