# Supplementary material for: Endolysosomal inhibition uncouples tau uptake from intracellular seeding
Source: J Biol Chem. 2026 Aug 27;302(10):113498. doi: 10.1016/j.jbc.2026.113498 (PMC13671035; doi:10.1016/j.jbc.2026.113498)
Supplement: Supplementary file 6 [file mmc6.pdf]

Figure S3

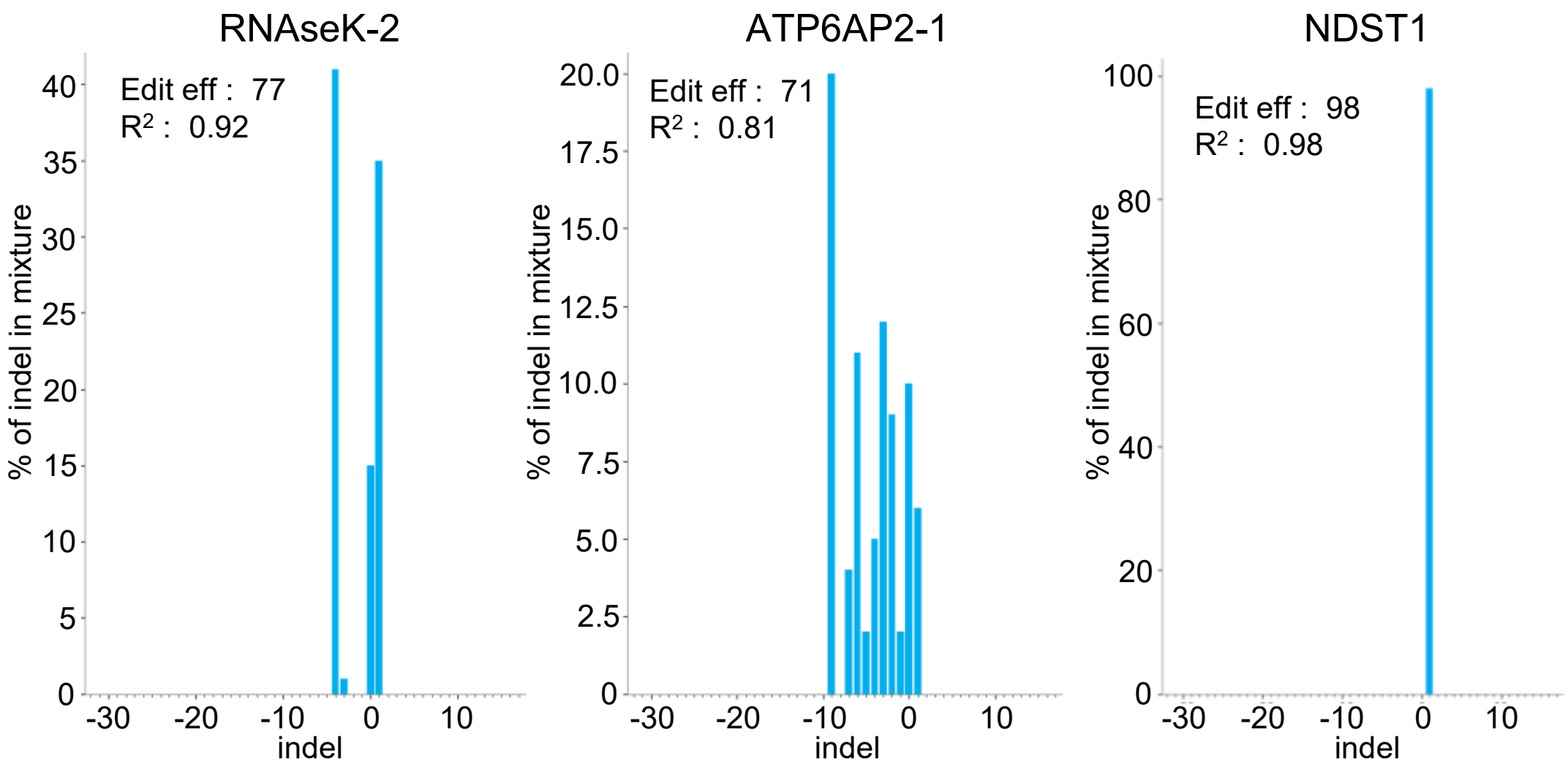

RNAseK-2
Edit eff : 77
R^2 : 0.92
% of indel in mixture
indel
ATP6AP2-1
Edit eff : 71
R^2 : 0.81
% of indel in mixture
indel
NDST1
Edit eff : 98
R^2 : 0.98
% of indel in mixture
indel
