# Supplementary material for: Endolysosomal inhibition uncouples tau uptake from intracellular seeding
Source: J Biol Chem. 2026 Aug 27;302(10):113498. doi: 10.1016/j.jbc.2026.113498 (PMC13671035; doi:10.1016/j.jbc.2026.113498)
Supplement: Supplementary file 7 [file mmc7.pdf]

Figure S4

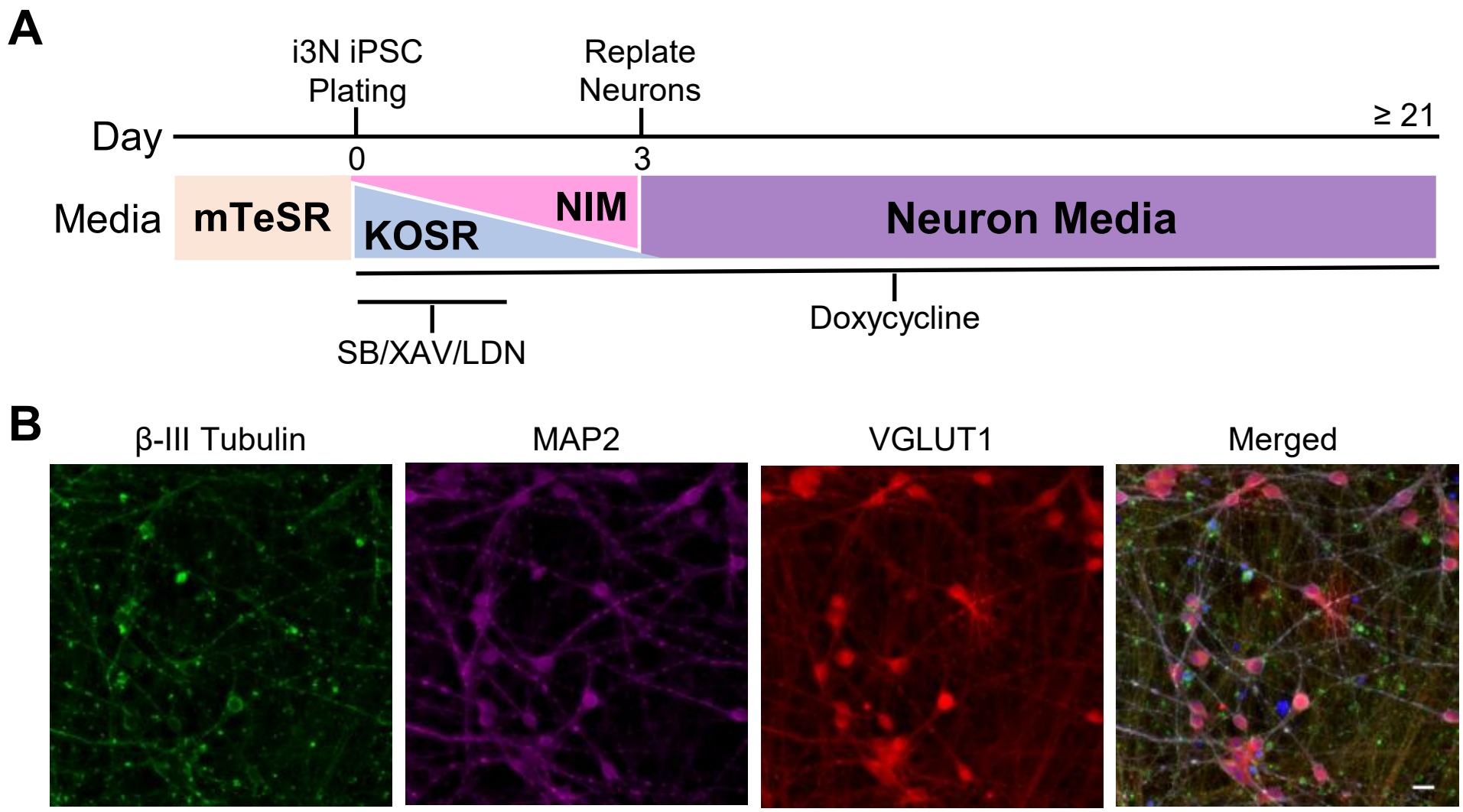
A
i3N iPSC
Plating
Replate
Neurons
Day
0
3
≥ 21
Media
mTeSR
KOSR
NIM
Neuron Media
Doxycycline
SB/XAV/LDN
B
β-III Tubulin
MAP2
VGLUT1
Merged
