# Supplementary material for: Endolysosomal inhibition uncouples tau uptake from intracellular seeding
Source: J Biol Chem. 2026 Aug 27;302(10):113498. doi: 10.1016/j.jbc.2026.113498 (PMC13671035; doi:10.1016/j.jbc.2026.113498)
Supplement: Supplementary file 8 [file mmc8.pdf]

Figure S5

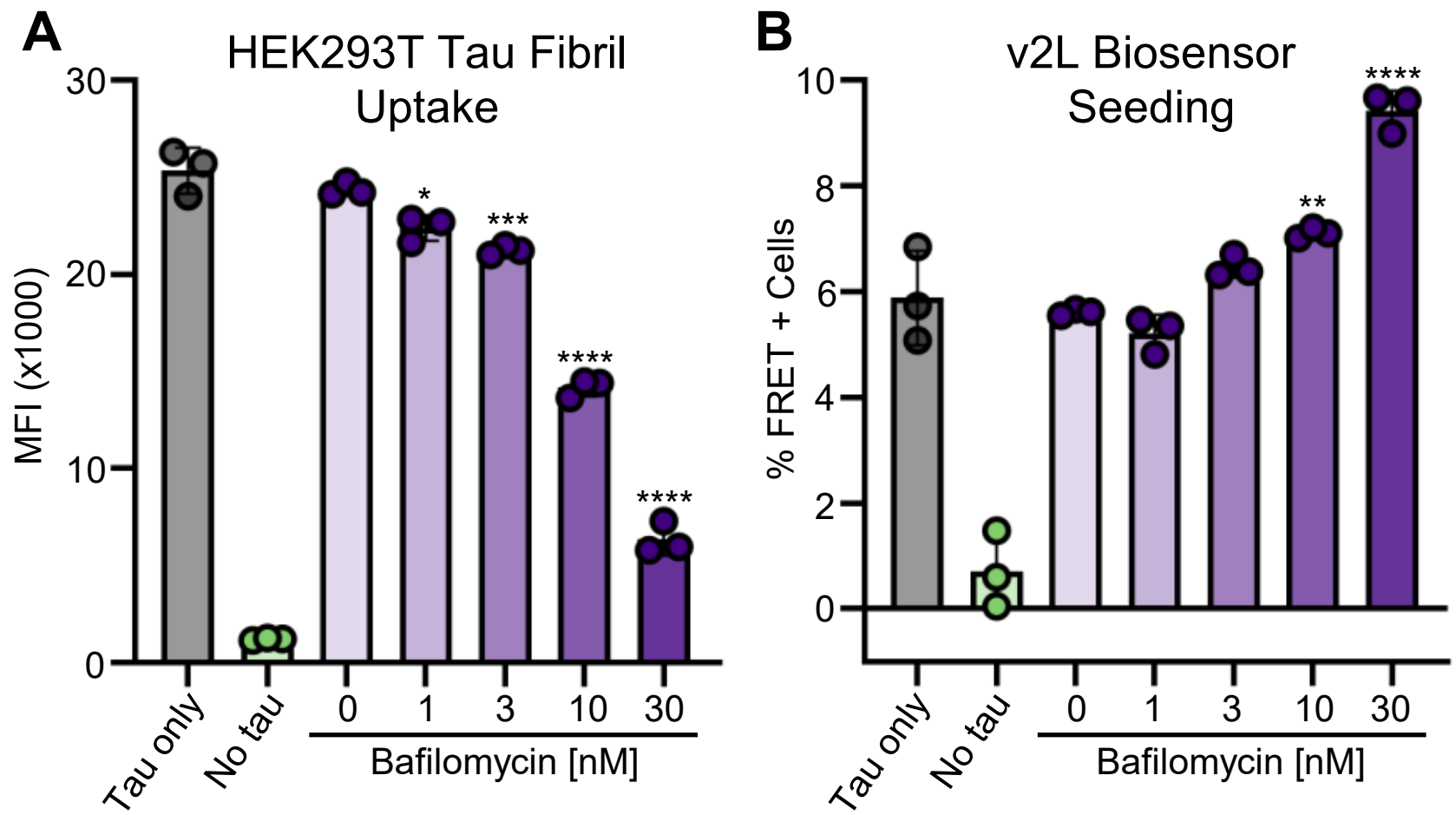

A
HEK293T Tau Fibril Uptake
MFI (x1000)
30
20
10
0
*
***
****
****
Tau only
No tau
0
1
3
10
30
Bafilomycin [nM]
B
v2L Biosensor Seeding
% FRET + Cells
10
8
6
4
2
0
**
****
Tau only
No tau
0
1
3
10
30
Bafilomycin [nM]
