# Supplementary material for: Endolysosomal inhibition uncouples tau uptake from intracellular seeding
Source: J Biol Chem. 2026 Aug 27;302(10):113498. doi: 10.1016/j.jbc.2026.113498 (PMC13671035; doi:10.1016/j.jbc.2026.113498)
Supplement: Supplementary file 9 [file mmc9.pdf]

Figure S6

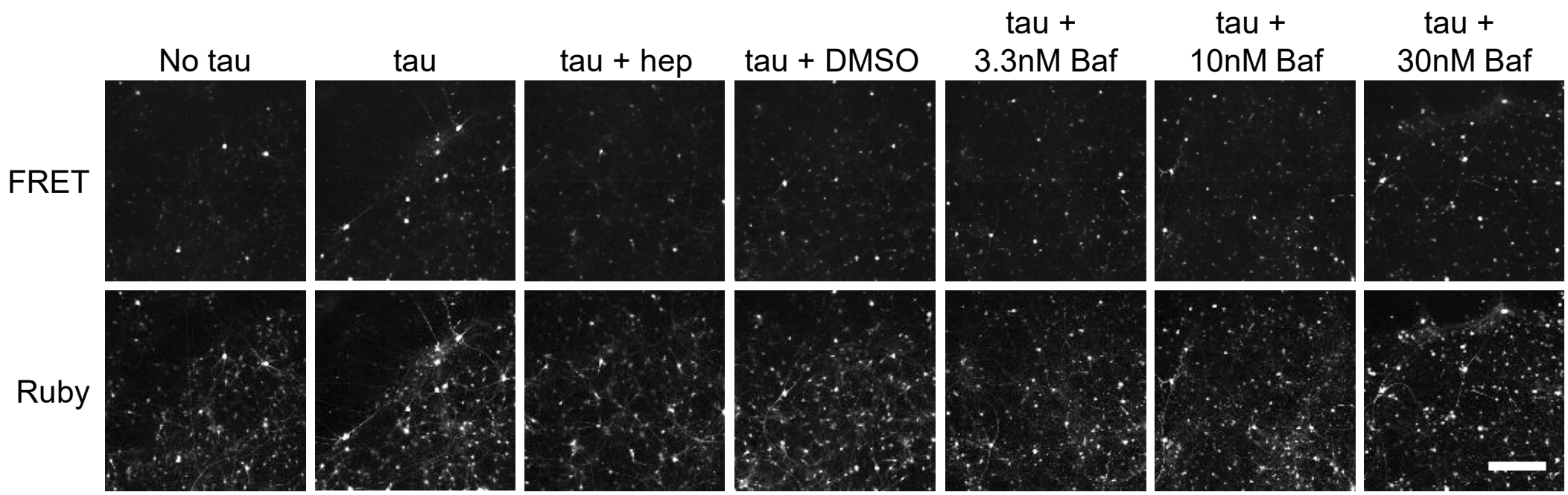

No tau
tau
tau + hep
tau + DMSO
tau + 3.3nM Baf
tau + 10nM Baf
tau + 30nM Baf
FRET
Ruby
