# Supplementary material for: Endolysosomal inhibition uncouples tau uptake from intracellular seeding
Source: J Biol Chem. 2026 Aug 27;302(10):113498. doi: 10.1016/j.jbc.2026.113498 (PMC13671035; doi:10.1016/j.jbc.2026.113498)
Supplement: Supplementary file 10 [file mmc3.docx]

Supporting Information Figure Legends

**Figure S1. Tau fibrils and concentration optimization.** A, Electron microscopy image of the 2N4R tau fibrils used in the paper. Scale bar = 0.5mm. B, Tau fibrils at different concentrations (monomer equivalent) were added to biosensor cells for 48 h. Cells were trypsinized and analyzed for FRET. The experiment was repeated at least two times with similar results. Data represent mean ± SD of technical replicates.

**Figure S2. Representative of one duplicate from cells during CRISPR screen of Gecko library A.** A, Cells not treated with tau fibrils were run as a control to set up the negative gate. B, Cells run during the first, faster sort. The 0.81% of A647 negative cells were collected. C, The second, slower sort of the collected cells from the first sort. In this sort 20.1% of A647 negative cells were ultimately collected for processing.

**Figure S3. ICE analysis of CRISPR KOs**. HEK293T cells were transduced with lentivirus to knockout RNAseK and ATP6AP2 and kept in puromycin for 8 days. Genomic DNA was amplified by PCR for regions around the CRISPR cut site. Sanger sequencing was performed and analyzed by the Synthego ICE CRISPR analysis tool. Indel analysis shows the edited gene locus with the predicted range of insertions and deletions.

**Figure S4. Differentiation and characterization of iPSC-derived cortical neurons.** A, Schematic of the differentiation of i^3^N iPSCs into glutamatergic cortical neurons. B, iPSC-derived neuronal cultures (day 35 post-differentiation) co-stain for neuronal markers b-III tubulin and MAP2 as well as the marker for glutamatergic neurons VGLUT1. Scale bar = 20mm.

**Figure S5. Uptake and seeding assays with only a 1h pre-incubation of bafilomycin**. A, HEK293T cells were incubated with varying concentrations of bafilomycin for one hour. Cells were washed and tau fibrils were added for four hours. Cells were then harvested and run on flow cytometry. B, Biosensor cells were incubated with varying concentrations of bafilomycin for one hour. Cells were washed and tau fibrils were added. Cells were harvested two days later and run on flow cytometry. All experiments were repeated at least two times with similar results. Data represent mean ± SD of technical replicates. Statistical analysis was performed using one-way ANOVA with Dunnett’s multiple-comparisons test compared to 0nM Bafilomycin; *p < 0.05, **p <0.01, ***p <0.0005, ****p <0.0001.

**Figure S6. Representative images of iPSC-derived neurons from the experiment in Fig. 3D showing FRET and tauRD-Ruby**. Scale bar = 40mm.

**Figure S7. Methyl-beta cyclodextrin treatment of HEK293T cells for 4 hours and 30min.** HEK293T cells were incubated in serum-free media with freshly made MBCD for 4.5 hours. Viability was tested by Cell-Titer-Glo. Data represent mean ± SD of technical replicates.

**Figure S8. Degradation of Tau-RD or Tau-full-length from TET-OFF cells.** A, Tet-OFF cells expressing Tau-RD (without seeding or constitutively seeding) or full-length tau with YFP or clover fusions were plated on two separate 96 well plates the day before the experiment. The day of the experiment one plate was placed at 4 °C and another at 37^o^C for 4 hours. They were then placed at 37 °C for varying times. Cells were harvested and analyzed by flow cytometry for fluorescence. Data represent mean ± SD of technical replicates. B, Before harvesting the cells in A) were imaged on a confocal microscope. Scale bar = 100mm.

**Figure S9. Analysis of tau immunoprecipitated from AD1 lysate.** A, Control protein or lysate was loaded on a SDS-PAGE as well as lysate before immunoprecipitation (Pre-IP), lysate immunoprecipitated with a control mouse IgG, lysate immunoprecipitated with anti-tau HJ8.5 antibody, and the supernatants from both of these immunoprecipitations. B, Tau immunoprecipitated from an Alzheimer’s disease brain lysate by HJ8.5 anti-tau Ab or recombinant tau fibrils were exposed to v2L biosensor cells at 4 °C or 37 °C for 4 h. Cells were then trypsinized and replated. After 48 h cells were harvested and intracellular aggregation was determined by FRET flow cytometry. Experiments were repeated at least two times with similar results. Data represent mean ± SD of technical replicates. Statistical analysis was performed using two-way ANOVA with Dunnett’s multiple-comparisons test compared to no tau; *p <0.005, **p < 0.005, ****p <0.0001.

**Table S1. Genome-wide CRISPR screen results.** Spreadsheet containing the combined, complete MAGeCK analyzed results sorted by positive selection p-value for the tau fibril uptake CRISPR screen in HEK293T cells using the human GeCKO full-genome library.
